# Epigenetic disruption of the RARγ complex impairs its function to bookmark AR enhancer interactions required for enzalutamide sensitivity in prostate cancer

**DOI:** 10.1101/2023.12.15.571947

**Published:** 2023-12-19

**Authors:** Sajad A Wani, Shahid Hussain, Jaimie S Gray, Debasis Nayak, Hancong Tang, Lillian M Perez, Mark D Long, Manjunath Siddappa, Christopher J McCabe, Lara E Sucheston-Campbell, Michael R Freeman, Moray J Campbell

**Affiliations:** 1 Division of Pharmaceutics and Pharmacology, The Ohio State University, Columbus, OH 43210; 2 Division of Cancer Biology, Cedars Sinai Cancer; 3 Board of Governors Innovation Center, Department of Biomedical Sciences, Cedars-Sinai Medical Center, Los Angeles, CA 90048; 4 Division of Cancer Therapeutics, Cedars Sinai Cancer, Departments of Urology and Biomedical Biomedical Sciences, Cedars-Sinai Medical Center, Los Angeles, CA 90048; 5 Roswell Park Comprehensive Cancer Center, Elm and Carlton Streets, Buffalo, NY 14263; 6 Institute of Metabolism and Systems Research (IMSR), and Centre of Endocrinology, Diabetes and Metabolism (CEDAM), University of Birmingham, Birmingham, UK; 7 Department of Cancer Prevention and Control, Cedars-Sinai Medical Center, Los Angeles, CA 90048

## Abstract

Lineage plasticity in advanced prostate cancer (PCa) involves a corruption of differentiation programs, often regulated by the androgen receptor (AR). This study explores an under-explored cistromic mechanism: how type II nuclear receptors like RARγ may affect AR control of prodifferentiation transcriptional programs. The components of the RARγ complex are dynamic in PCa cell models and enriched for miR-96 targets, partly due to m6A-marked miR-96 recognition elements. Restoration of TACC1 and RARγ, both miR-96 repressed targets, in 22Rv1 cells augmented the AR cistrome, particularly in active enhancers and super-enhancers. In 22Rv1 cells the RARγ complex was enriched for bookmarking components, and created nucleosome-free chromatin enriched for H3K27ac, and profoundly enhanced the dihydrotestosterone (DHT)-dependent AR cistrome in G2/M cells and are all indicative of bookmarking functions. The RARγ-dependent and DHT-regulated AR cistrome-transcriptome relationships were significantly strengthened, notably in Active Enhancers, associated with AR-dependent luminal differentiation transcriptional programs. Interestingly RARγ-dependent AR cistromes significantly overlapped with ONECUT2, but the comparative transcriptional analyses supported an antagonistic function between RARγ and ONECUT2. Finally, the footprint of these data was detected in advanced tumors. For example, the miR-96 targetome and RARγ/ONECUT2 antagonized genes were significantly similar to AR signaling inhibitor-induced alternative lineages and metastatic tumors. Partial correlation analyses revealed RARγ CoAs identified by RIME, including SSRP1, significantly strengthened the correlations between AR and RARγ-TACC1-dependent DHT-regulated target genes in SU2C advanced PCa, and differentially-expressed genes between tumors with high RARγ and low ONECUT2 expression compared to the reverse was significantly associated with elevated AR score. In summary, the RARγ complex, selectively targeted by miR-96 functions as an enhancer re-programmer by bookmarking chromatin in G_2_/M cells to sustain and restrict AR transcriptional competency required for programs such as luminal differentiation, and antagonizing ONECUT2.

## INTRODUCTION

Patients with advanced prostate cancer (PCa) receive androgen receptor (NR3C4/AR) signaling inhibitors (ARSI), such as such Enzalutamide (Enza), which are initially successful recurrent PCa frequently emerges (reviewed in(1). Acquisition of therapy resistance is frequently driven by altered AR functions, including enhancer switching to novel gene regulatory regions(2) that favor growth promotion instead of differentiation(3–9) and the acquisition of alternative lineages, such as neuroendocrine (NE) PCa. Identifying the mechanisms that allow this shift cell fate may allow novel routes to restore luminal phenotypes, perhaps by targeting stem cell-like components, and may make tumors more amenable to therapy(10). Arguably, this is the goal of Bipolar Androgen Therapy for PCa, in which the non-malignant features of AR signaling are sustained to support luminal differentiation(11).

The AR interacts with multiple other nuclear receptors (NRs) and coregulators, which collectively form a genomic network to sense a wide range of lipophilic ligands and signal transduction inputs to regulate cell fate decisions, metabolism, and other biological processes (reviewed in(12–14)). Type I NRs, such as the AR, bind ligand with high affinity, which leads to nuclear translocation and target gene regulation. By contrast, Type II NRs such as retinoic acid receptor gamma (NR1B3/RARG, encodes RARγ) are nuclear resident, independent of ligand, and instead ligand exposure leads to genomic redistribution and modulation of target genes. Prominently, the AR in the normal prostate gland promotes luminal phenotypes, survival and can promote proliferation (15), whereas other NRs such as RARγ promote prostate growth restraint (16,17).

There are well-studied examples of how epigenetic mechanisms re-shape the genomic capacity of the AR to favor proliferative gene expression programs, for example, as a result of changes in expression of AR-interacting coregulators, such as the coactivator CBP/P300(18,19) and the corepressors NCOR1 and NCOR2(20–22). Similarly, microRNAs (miRNAs) also govern elements of this network, for example by targeting the pioneer factor FOXA1(23) and other lineage identity drivers. More widely, genomic analyses in PCa progression revealed there are many infrequently mutated chromatin remodelers, the so-called “long-tail” mutant epigenetic drivers(24). Underscoring the cellular impact of altered transcriptional processes, recent studies suggest that luminal-type features can be restored by targeting the NR-pioneer factor FOXA2(25) and other coregulators govern Enza sensitivity(21). Indeed, genomic(8,26) and epigenomic(27–29) events alter expression of a relatively small group of transcription factors (TFs), perhaps fewer than 100(30) including ONECUT2, a master regulator of metastasis and drug resistance in PCa(31). Collectively, these altered coregulators and TFs shape AR-genome interactions and sensitivity to both endogenous hormone and ARSIs.

There is extensive crosstalk between NRs, and therefore their genomic distribution patterns are often shared and can impact gene regulation in a combinatorial manner. For example, NR-genomic interactions can converge on complex genomic sites such as high occupancy target (HOT) regions (32–34). Outside of NR biology, specific complexes have been established, for example in pluripotent cells, to sustain nucleosome positioning during mitosis to flag genomic sites required for post-mitotic immediate transcriptional reactivation (35–39). For example, SMARCB1 and SMARCA4/BAF are members of the BRG1/BRM-associated factor complex and display selective binding in mitotic embryonic stem cells (40). There is some evidence to suggest that nuclear-resident Type II NRs can also impact gene regulation mechanisms by bookmarking genomic sites on mitotic chromatin (35,39,41–44). The orphan receptors NR3B2/ERR2 and NR5A2/LRH-1(45) can bookmark genomic binding sites during mitosis for other transcription factors to bind subsequently(42,46–48). NR-mediated bookmark mechanisms have not been investigated in either PCa or cancer generally and could add further to understanding of crosstalk mechanisms between NRs.

We previously showed that RARγ was significantly down-regulated down-regulated in 60% of localized PCa tumors in the TCGA cohort compared to non-malignant samples(49), in part by miR-96–5p (miR-96) which targets RARG and a known coactivator TACC1 in non-malignant prostate cells. This mechanism impacted the DHT-dependent sensitivity of AR to regulate genes in PCa cell lines, and surprisingly that the RARγ binding sites were enriched for the ONECUT2 motif (50).

**Figure 1:**
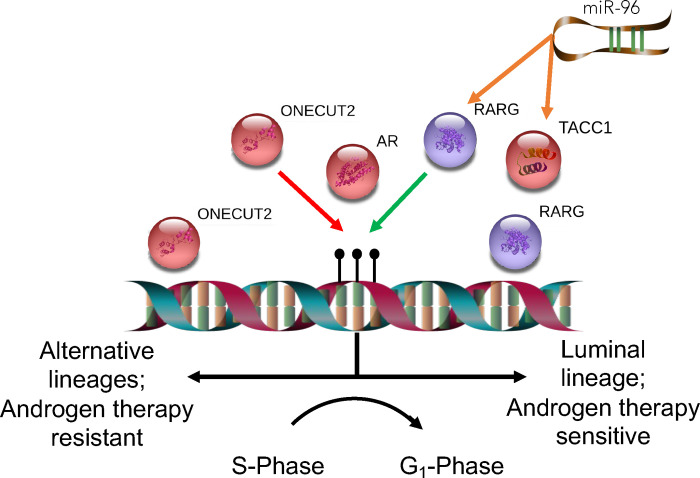
Graphical abstract of workflow

In the current study we sought to define the impact of the RARγ complex in advanced PCa through three broad approaches (Figure 1; **Graphical Abstract**). Firstly, we undertook proteomic approaches to define the RARγ complex across PCa models, and given there are a wide number of identified miR-96 targets (51–56), we sought to define the prominence of the RARγ complex as a miR-96 target, and to test how the epi-modification, N^6^-methyladenosine (m6A)(57–60), shaped the miR-96 targetome. Next, we examined how RARγ and TACC1 shaped the AR cistrome, and we tested the possibility that RARγ could function to bookmark the AR cistrome and dictate the AR-dependent transcriptional response to DHT and Enza. Finally, we tested if the RARγ complex could antagonize the master regulator ONECUT2 to promote a luminal transcriptional programs and Enza responsiveness.

These approaches support a model whereby the RARγ complex functions to bookmark AR-dependent luminal transcriptional programs and antagonize ONECUT2, and miR-96 targeting corrupts these functions allowing enhancer re-programming that regulates the sensitivity towards Enza.

## MATERIALS AND METHODS

### Reagents.

DHT(Sigma), CD437 (Sigma), Enza (Sigma) mir-96 mimic and antagomir (Ambion, nocodazole (CST)

For Western immunoblots the following antibodies were used; Anti-RARγ (D3A4) rabbit mAb #8965 Cell Signaling Technology (CST); Anti-Myc Tag (9B11); Anti-TACC1 mouse mAb #2276 (CST); Anti-AR-V7 rabbit mAb #ab198394 (Abcam); Anti-AR (D6F11) rabbit mAb #5153 (CST); Anti-PGC-1α (3G6) Rabbit mAb #2178 (CST); Anti-REG4 Rabbit Polyclonal Antibody #43411 (CST); Anti-PMEPA1 rabbit polyclonal antibody # 16521–1-AP; Anti-ERRg rabbit mAb (EPR8100) #ab128930 (Abcam); Anti-DLC1 rabbit polyclonal antibody #PA5–53635 (Invitrogen); anti-PCNA rabbit polyclonal antibody #ab15497 (Abcam).

For rapid immunoprecipitation mass spectrometry of endogenous protein (RIME) assay RARγ (D3A4) #8965 CST antibody was used.

For CUT&RUN the following antibodies were used; Anti-Acetyl-Histone H3K27 (D5E4) XP^®^ rabbit mAb #8173 (CST); anti-GFP antibody, (ChIP Grade-ab290, Abcam); anti-AR (D6F11) XP^®^ rabbit mAb #5153 (CST), anti-IgG #2729S (CST), rabbit anti-H3S10ph #39254 (Active Motif).

### Biological Resources.

Prostate cancer cell lines LNCaP and 22Rv1 were obtained from the ATCC and maintained in RPMI-1640 media (Gibco) supplemented with 10% FBS and penicillin/streptomycin. Non-malignant HPr1AR cells were maintained in keratinocyte serum free medium (SFM; Gibco) supplemented with penicillin/streptomycin/gentamicin(61). Cells were screened for mycoplasma every 2 months (Mycoplasma PCR Detection Kit (Sigma Aldrich) and authenticated regularly by short tandem repeat profiles at The Ohio State University Comprehensive Cancer Center Genomics Shared Resource.

### Lentiviral constructs and transduction.

pLenti-CMV-RARγ-C vector (GFP) and pLenti-CMV-TACC1-C vector (MYC) and pLenti-III-Blank vector were custom synthesized (Applied Biological Materials). Lentiviruses MOI were produced as per manufacturer’s protocol(62) and transfected into cells followed by positive selection with puromycin (1 μg/mL (Sigma)). TACC1 and RARγ expression was confirmed by qPCR and Western immunoblot. These 22Rv1 variants are specifically 22Rv1 cells stably transfected with an empty vector (22Rv1-mock) or a GFP-tagged RARγ (22Rv1-RARγ). 22Rv1-RARγ-TACC1 cells were generated by transient transfection of TACC1 into 22Rv1-RARγ cells; we demonstrated TACC1 protein expression was sustained for ~72h.

### Cell viability.

Cells were plated in 96-well, white-walled plates to ensure exponential growth were plated and dosed at 0 h, as previously(50). At the indicated time points cellular ATP was measured using CellTiter-Glo^®^ (Promega) and luminescence detected with Synergy^™^ 2 multi-mode microplate reader (BioTek^®^ Instruments). Each experiment was performed in at least triplicate wells in triplicate experiments.

### Clonogenic assays.

Cells were plated in 6 well plate and treated as indicated every three days for 14 days and colony formation was measured by crystal violet stain and quantified(63).

### Apoptosis Assay.

Cells were treated with agent alone or with miR-96 inhibitor or scramble control (as below), treated with Annexin-V-FLUOS (Roche Diagnostics) and analyzed by flow cytometer (Becton Dickinson).

### Cell cycle measurements.

Cells were treated as for apoptosis, harvested, stained with PI and incubated (45 min in the dark) and cell cycle profiles analyzed on a flow cytometer (Becton Dickinson).

### Western Immunoblotting.

Cells were harvested and lysed in ice cold RIPA buffer containing Protease inhibitors (Roche). Equal amounts of protein (30–60μg) were resolved via SDS polyacrylamide gel electrophoresis (SDS-PAGE) using precast 4–20% gradient polyacrylamide gels (Bio-Rad). Blocked membranes were probed with primary antibody for 3 hours at room temperature and subsequently for 1 hour species-appropriate secondary antibody at room temperature using enhanced chemiluminescence substrate (Pierce). Signal quantification was performed using the Protein Simple Fluorochem M Imager.

### RT-qPCR.

Total RNA was isolated (TRIzol^®^ reagent (Thermo Fisher Scientific)) and complementary DNA (cDNA) prepared (iScriptTM cDNA Synthesis Kit (Bio-Rad)) and relative gene expression quantified via Applied Biosystems 7300 Real-Time PCR System (Applied Biosystems), for both TaqMan and SYBR Green (Thermo Fisher Scientific) applications. All SYBR Green primers were tested for specificity by melting curve analysis. Fold changes determined using the 2-ΔΔCt method. Gene expression master mix (Applied Biosystems) and the TaqMan assays RARγ (Hs01559230_m1), TACC1 (Hs00180691_m1), TWF1 (Hs00702289_s1), HMGA2 (Hs04397751_m1), MYC (Hs04397751_m1) were used to measure expression levels. 18s (Hs99999901_s1) and GAPDH (Hs02786624_g1) were used for normalization.

### Transfection of miRNA mimic and antagomir.

Transient transfection of miRNA mimic/inhibitor (50nM) (Ambion) was undertaken using Lipofectamine^®^ 2000 in presence of Opti-MEM Reduced Serum Medium (Thermo fisher Scientific). Concentrations of miRNA mimic/inhibitor and transfection reagents were optimized using BLOCK-iTTM Alexa Fluor^®^ Red Fluorescent Control (Ambion) as well as by mirVana^™^ miRNA mimic miR-1 positive control and mirVana^™^ miRNA Inhibitor, let-7c positive control. Expression of TWF1 in miR-1 mimic positive control transfected and HMGA2/MYC in let-7c inhibitor positive control transfected was detected by quantitative polymerase chain reaction (qPCR) to optimize transfection conditions.

### Identification of MRE by pull-down and alignment of captive transcripts—sequencing (IMPACT-Seq).

IMPACT-Seq(64) was undertaken on 5×10^6^ cells transfected with 3’ biotinylated miRIDIAN miR-96 mimic and scramble (50 nM, 24h and 48h) (Dharmacon, Inc.) using Lipofectamine 2000. RNA was then extracted from washed beads using Trizol LS and treated with T4 Polynucleotide Kinase (New England Biolabs) to obtain 5’ phosphate ends for subsequent ligations and passed through NucAway columns (Ambion) to remove RNAs <20 nt in length. Small RNA libraries were generated using NEBNext Multiplex Small RNA Library Prep (New England Biolabs) following the manufacturer’s recommendations for 100ng input material. PCR-amplified small RNA libraries were size selected to 130–200 bp fragments. Libraries characterized on an Agilent Tape Station on a DNA1000 High-Sensitivity Screen Tape and quantified by Qubit were pooled and sequenced at 75 bp read length on the MiSeq platform.

### m6A Sequencing:

m6A seq was performed on 20×10^6^ cells using EpiMark N6-methyladenosine enrichment kit (New England Biolabs). RNA-seq libraries prepared from eluate and the input using Total RNA Stranded RNA library prep (New England Biolabs) and was checked for fragment length using the Agilent 2100 Bioanalyzer. The libraries were sequenced at 50 bp read length and were sequenced on an NovaSeq SP with a target of ~40M PE reads per sample.

### Rapid immunoprecipitation mass spectrometry of endogenous protein (RIME).

RIME(65) was undertaken in presence or absence of DHT (10nM, 6h), CD437 (400nM, 6h). 20×10^6^ cells were crosslinked with 1% formaldehyde solution, quenched with glycine (0.1M, pH 7.5) and harvested in cold PBS. Nuclei were separated and subjected to sonication (30s on 30s off cycles for 30mins) for genomic DNA fragmentation. Nuclear proteins were separated using 10% triton-X with high-speed centrifugation. RARγ or IgG antibody conjugated beads were incubated with nuclear lysates overnight and washed ten times with RIPA buffer and 2 washes of Ambic solution. LC-MS/MS was performed over a 2-hour separation and mean spectral count data captured.

### Assay for Transposase-Accessible Chromatin using sequencing (ATAC-Seq).

Omni-ATAC-seq(66) was undertaken in 5×10^4^ cells treated for 6h with DHT (10nM) or CD437 (400 nM), and resuspended in 50ul of ATAC-resuspension buffer, nuclei pelleted in ATAC-wash-resuspension buffer and resuspended in transposition mix (2X TD buffer, 1X PBS, Digitonin 0.01%, tween 20 0.1%, NFW 5ul, and Illumina transposase 2.5ul). Libraries were PCR-amplified using the NEBNext Hi-Fidelity PCR Master Mix and Integrated DNA Technologies (IDT) 8 bp unique dual indexing (UDI) adapters. The PCR cycle number were optimized as indicated by qPCR fluorescence curves. Primer dimers and large >1,000 bp fragments were removed using AMPure XP beads (Beckman Coulter). The quality of libraries was checked on Agilent High Sensitivity DNA Bioanalysis and quantified by qubit. Libraries were sequenced on NovaSeq6000 S1 PE150bp (v1.5) with 54M-66M reads per sample.

### Cleavage Under Targets and Release Using Nuclease (CUT&RUN).

CUT&RUN(67) was undertaken with antibodies to GFP (for RARγ), AR, H3K27ac, H3S10P and IgG. Briefly, 0.5×10^6^ cells were treated for 6h with DHT (10nM) or CD437 (400 nM). For TACC1, the pLenti-CMV-TACC1-C term RFP vector was transiently overexpressed for 48h followed by similar drug treatments. Harvested cells were washed with pre-activated Concanavalin A-coated beads mixed with antibody overnight at 4°C. CUTANA pAG-MNase (EpiCypher) was incubated with sample for 10 minutes at room temperature. Fragmented DNA samples was purified using the Monarch DNA Clean up Kit (New England Biolabs). The libraries were prepared from 5–10ng purified CUT&RUN-enriched DNA, using the NEB Ultra II Library Prep Kit (New England Biolabs) per manufacturer’s instructions and sequenced using Hiseq 4000 PE 150bp. Transcription factor modifications for sub-nucleosomal size (<120bp) DNA fragments were undertaken as per protocol.

### RNA-Seq.

We undertook RNA Seq in three experimental settings. 1. In the presence of miR-96 mimic/scramble (50nM) by transient transfection in HPr1-AR, LNCaP and 22Rv1 cells and RNA harvested at 48h. 2. In 22Rv1-RARγ and 22Rv1-Mock cells treated with DHT (10nM) or CD437 (400nM) for 24h. 3. 22Rv1 cells were treated with the combination of ENZA and miR-96 inhibitor as above. Total RNA was extracted E.Z.N.A.^®^ Total RNA Kit I, Omega Bio-tek^®^) and subjected to DNase treatment and purification by the RNA Clean & Concentrator kit (Zymo Research) to yield high-quality RNA for library preparation. RNA-seq libraries were prepared using Illumina’s TruSeq Stranded protocol. Quality of libraries were determined via Agilent 4200 Tapestation using a High Sensitivity D1000 Screen Tape assay and quantified by KAPA qPCR (KAPA BioSystems). Approximately 60–80 million paired-end 150 bp sequence reads were generated for each library on the HiSeq 4000 platform.

### Label free quantitative proteomics (LFQ).

MiR-96 mimic/scramble (50nM) was transiently transfected into HPr1-AR, LNCaP and 22Rv1 cells for 48h and lysed with 5% SDS in 50mM ammonium bicarbonate solution. Protein concentration was measured and 70μg of protein was used for S-Trap Digestion. Capillary-liquid chromatography-nanospray tandem mass spectrometry (Capillary-LC/MS/MS) of protein identification was performed on a Thermo Scientific orbitrap Fusion mass spectrometer.

#### Data analyses and integration.

All sequencing was performed at the Nationwide Children’s Hospital Institute for Genomic Medicine and FASTQC files were QC processed and aligned to Hg38 (Rsubread)(68).

### IMPACT Seq and m6A Seq.

Differentially enriched miR-96 binding sites and m6A were identified with csaw (69) and annotated to genes (ChIPpeakAnno)(70), and only those retained that directly overlapped with a transcript.

### RIME and LFQ.

Capillary-liquid chromatography-nanospray tandem mass spectrometry (LC-MS/MS) was acquired over a 2-hour separation using a data-dependent MS/MS approach at a resolving power to achieve high mass accuracy MS determination. Label free shotgun proteomics was used to eliminate nonspecific interactors(71) and the mean spectral count results generated by two protein inference engines (Epifany (72) and FIDO (73)). This count matrix was processed in an edgeR workflow to identify differentially enriched proteins, visualized with volcano plots and network enrichment analyses (e.g. stringdb). Identified proteins were annotated enriched proteins by functional class (coactivator (CoA), corepressor (CoR), Mixed function coregulator (Mixed) or transcription factor (TF))(74). Hypergeometric tests were used to test enrichment for CoA, CoR, Mixed, TF, “long-tail” mutant epigenetic drivers(24), or miR-96 bound and regulated target genes, with or without m6A modification, in each RARγ complex.

### CUT&RUN.

Cistromes were analyzed with csaw with appropriate window sizes for either TF (AR and RARγ) or larger ones for H3K27ac. Significantly differentially enriched regions (FDR < .1) were overlapped with ChromHMM(75) derived underlying epigenetic states in LNCaP (ref), and also mined for TF motif analyses (MotifDb).

In order to find potential transcription factor binding enrichment within cistromes, GIGGLE was utilized to query the complete human transcription factor ChIP-seq dataset collection (10,361 and 10,031 datasets across 1,111 transcription factors and 75 histone marks, respectively) in Cistrome DB(76). Prostate specific filtering limited analysis to 681 datasets across 74 TFs and 238 datasets across 19 HMs. For each query dataset, the overlap with each experimental cistrome was determined. Putative co-enriched factors were identified by assessment of the number of time a given factor was observed in the top 200 most enriched datasets relative to the total number of datasets for that factor in the complete Cistrome DB (> 1.2 FC enrichment over background). For prostate specific analysis, overlaps across datasets were averaged for each factor.

H3K27ac cistromes were analyzed for super-enhancers by combining with BRD4 cistrome from 22Rv1(77,78) using the ROSE algorithm(79).

### ATAC-Seq.

ATAC-Seq data were separated into nucleosome free (NF), mono-, di- and tri-nucleosome compartments (ATACSeqQC)(80) and analyzed with csaw.

### RNA-Seq.

Transcript abundance estimates were normalized and differentially expressed genes (DEGs) identified using a standard edgeR pipeline, and gene set enrichment analysis (GSEA) performed. For transcript-aware analyses, the FASTQ files were aligned with salmon(81) and differentially enriched transcripts were identified using DRIMSeq (82) in a similar workflow to edgeR.

### Cistrome-Transcriptome analyses.

To test the significance of the ATAC-Seq or CUT&RUN cistromes to transcriptomes we applied a modified BETA method(83). Specifically, within each cell type we summed significance of the peaks within 100 kb of each annotated DEG multiplied by the absolute fold change for the same DEG and weighted by the peak distribution (proximal versus distal), or unweighted. We defined this score as the weighted cistrome-transcriptome (wt-C-T) and tested the difference between controls and modified cell backgrounds using a Wilcox test.

### Data Availability:

IMPACT-, m6A-, CUT&RUN, RNA-, and ATAC-Seq data are available (GSE***)

## RESULTS

### The chromatin bound RARγ complex is enriched for coregulators, bookmarking factors, and RNA splicing factors.

**Figure 2.**
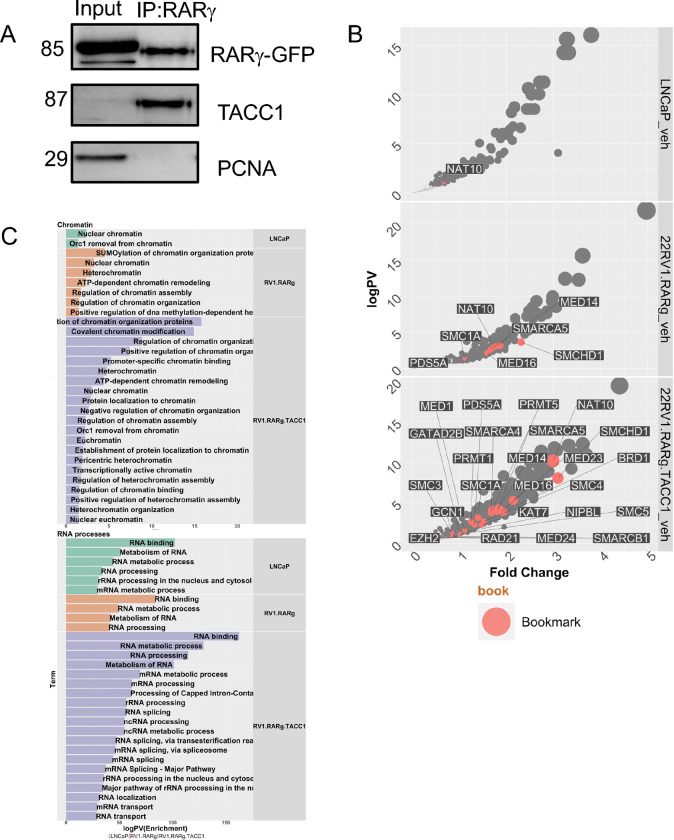
The RARγ complex in LNCaP and 22Rv1 cells. **A.** Co-immunoprecipitation of TACC1 with RARγ. **B.** Cells were treated with CD437 (400 nM, 6h) or vehicle control in quadriplicates and RIME analyses undertaken of RARγ in the indicated cells and significantly different proteins were identified using an edgeR workflow. Volcano plots depicting enrichment levels, compared to IgG controls, between the indicated cells in basal conditions and proteins associated with bookmarking (n=42) are shown in salmon. **C.** The positively and significantly enriched proteins in each complex were analyzed by StringDB and the enriched terms were grouped by most frequently enriched master terms (Chromatin, RNA Processes) and the significant terms illustrated as waterfall plots.

RARγ and TACC1 physically interact (Figure 2A), and to define the components in the complex we undertook RIME in LNCaP cells and 22Rv1 cells variants; 22Rv1 cells being a model of ARSI resistant PCa. As basal *RARG*/RARγ levels are very low in 22Rv1 cells we created a series of variants; 22Rv1-mock, 22Rv1-RARγ and 22Rv1-RARγ-TACC1, respectively. All cells were treated vehicle or the RARγ-selective ligand CD437 (400 nM, 6 h).

In each cell type several hundred RARγ-interacting proteins were significantly and positively enriched in the basal and CD437-treated state, which was greatest in 22Rv1-RARγ-TACC1 cells. The levels of RARγ enrichment was broadly comparable between LNCaP and 22Rv1 cells (Supplementary Figure 1) compared to IgG controls. However, in the basal state the number of RARγ-interacting proteins was greatest in 22Rv1-RARγ-TACC1 cells (n~580) in and least in LNCaP cells (n ~260).

Enrichment was tested for either coregulators or chromatin remodelers that are mutated in PCa, the so-called long-tail mutants(24) (Supplementary Figure 1A). Specifically, we examined enrichment of coactivators (CoAs), corepressors (CoRs), mixed function coregulators (Mixed) or transcription and mRNA stabilization factors (TFs)(74). There was significant enrichment of different groups of coregulators, for example mixed function coregulators were highly enriched in basal 22Rv1-RARγ-TACC1 cells (Supplementary Table 1A). There was also identification of canonical coregulators such as NCOR2 in basal LNCaP, and ligand treatment reduced the significance of enrichment. More widely the RARγ complexes were significantly enriched for proteins involved in other processes such as NSUN2, which is required for correct mRNA stabilization and translation mRNA, including for the AR itself (84) (Supplementary Figure 1A). Also of interest, several NRs were positively enriched in 22Rv1-RARγ compared to LNCaP including the AR itself, the orphan NRs NR2C1/TR2 and NR2C2/TR4, and RXRb and RXRα (Supplementary Table 1B). There was also enrichment of bookmarking factors in 22Rv1-RARγ and most clearly in 22Rv1-RARγ-TACC1 cells, including SMARCA4, SMARCA5, SMARCB1, and SMARCC1 (Figure 2B, salmon). Finally, the chromatin remodelers in the long-tail mutants(21) were most significantly enriched in basal 22Rv1-RARγ-TACC1 cells, and the most enriched factor was SMCHD1, which is participates in DNA repair and binding(85) (Supplementary Table 1C).

Network enrichment of RARγ-associated proteins was undertaken for GO and UniProt terms (Supplementary Figure 2A, 2B). Unsurprisingly, the most-enriched terms included Acetylation in basal and CD437-treated conditions. However, the frequency and significance of enrichment terms was greatest in 22Rv1-RARγ-TACC1 cells, suggesting RARγ and TACC1 together generates a functional complex. RNA binding and processing, including RNA splicing was frequent (Figure 2C). For example, the GO splice term (GO:0000398) were enriched across the cell models, and individual components were notable in the RARγ complexes including SART3 (Supplementary Figure 1B).

These data demonstrate that the RARγ complex is significantly enriched with coregulators known to interact with RARs, such as NCOR2, as well as other NRs, bookmarking factors, and more novel coregulators such as SSRP1, which is a member of the FACT chromatin remodeling complex(86). Splicing factors such as SART3 were enriched as well as splicing functions and RNA-processing.

### The RARγ complex is significantly enriched for miR-96 targets.

To test whether the RARγ complex is enriched for miR-96 targets we sought to determine if the cell background and time kinetics shaped the repertoire of miR-96:mRNA interactions and regulation of RNA and proteins. Specifically, we transfected a biotinylated miR-96 into either non-malignant prostate epithelial HPr1AR or LNCaP cells and isolated the bound mRNA after 24 and 48h by using IMPACT-Seq coupled with transcriptomic and proteomic analyses.

Under optimized transfection and miRNA-targeting conditions (Supplementary Figure 3), IMPACT-Seq revealed the miR-96 targetome was highly time- and cell-dependent. In HPr1AR cells more miR-96 recognition elements (MRE) was identified at 48h rather than 24h exposure to biotinylated miR-96 (n~7500), whereas this was reversed in LNCaP cells with more MRE identified at 24h (n~3225) than 48h. In HPr1AR the ~7500 MRE associated with ~2860 genes, and in LNCaP the ~3225 MRE associated with ~2380 genes (Supplementary Table 2A, B). There was only a modest overlap between HPr1AR and LNCaP of either MRE or targeted genes (Supplementary Figure 4A, B).

To define miR-96:RNA interactions further, we examined the MRE distribution on target genes. For protein-coding mRNAs in HPr1AR cells there was a pronounced 3’UTR bias for MRE at 24h whereas at 48h, when most MRE were detected, there was a comparable 5’UTR and 3’UTR enrichment. This was similar in LNCaP cells at 24h when most MRE were detected, there was also comparable 5’UTR and 3’UTR enrichment, however at 48h, with fewer MRE, there was a pronounced enrichment downstream of the 3’UTR. For non-coding RNA there was broadly comparable 5’UTR and 3’UTR enrichment between cells and time points, except for LNCaP cells at 48h where enrichment was pronounced in the body of the gene (Supplementary Figure 4C).

Enrichment of MRE with m6A revealed further distinctiveness of miR-96:RNA interactions. Again at the time points of most frequent miR-96:RNA interactions in each cell there was frequent overlap with m6A sites, although this was most significant in HPr1AR cells. Specifically, MRE and m6A sites significantly overlapped in HPr1AR at 48h (p=2.1e^−299^) and in LNCaP at 24h (p=1.8e^−14^). Again, in HPr1AR for coding RNA there was a 3’UTR bias of m6A-MRE and 5’UTR bias in LNCaP, but no difference for non-coding RNA (Supplementary Figure 4D).

**Figure 3:**
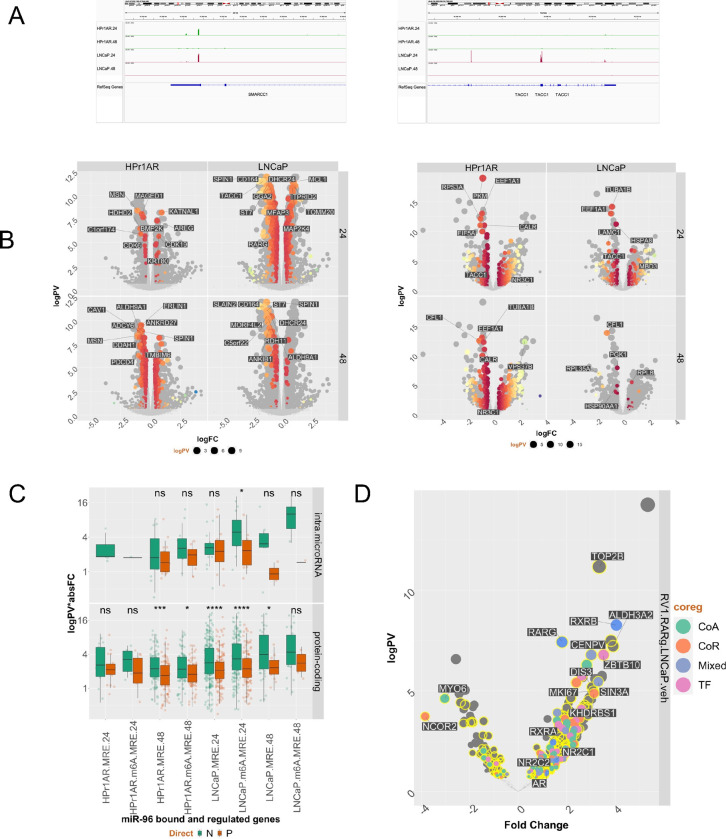
The miR-96 targetome and regulation of target genes. HPr1AR and LNCaP cells were treated with 3’ biotinylated miRIDIAN miR-96 mimic or scramble (50 nM, 24h and 48h) in triplicate and IMPACT-Seq undertaken. In parallel, in triplicate m6A-Seq was undertaken in vehicle treated cells. csaw was used to identify differentially enriched regions of miR-96 binding sites (MREs) and m6A sites, and the overlap of m6A-MRE was determined by bedtools. **A.** Representative genome browser views of MRE on SMARCC1 and TACC1 in HPr1AR and LNCaP cells. **B**. **Left.** RNA-Seq was undertaken in triplicate in HPr1AR and LNCaP cells transfected with miR-96 mimic or control for 48 h and BAM files were processed with edgeR workflow to identify significant differentially enriched genes (DEGs) (logPV > 1 & absFC > .37) and are illustrated on Volcano plots with DEGs that contained a MRE identified in the same cell background at either 24 or 48 h in spectrum colors for significance and top 10 most significant genes labelled. **Right.** Cells were treated as for RNA and label free quantitative proteomics undertaken and an edgeR workflow applied. **C**. MRE or m6A-MRE-containing genes were classified as protein-coding or protein-coding genes containing an intronic miRNA and difference tested of the absolute fold change between negatively and positively-regulated targets. **D.** Volcano plot of significantly differentially enriched proteins in basal 22Rv1-RARγ cells compared to LNCaP. Proteins were classified either as a Coactivator (CoA), Corepressor (CoR), Mixed function coregulator (Mixed) or transcription factor (TF) and whether they contained a MRE (yellow).

Considering the type of RNA bound by miR-96 further illustrated the specificities of miR-96:RNA interactions. For example, calculating the frequency of either MRE or m6A-MRE interactions with different types of RNA revealed that miRNA and protein-coding genes containing miRNA were more frequently targeted in HPr1AR than LNCaP (Supplementary Figure 4E). Similarly, categorization of miR-96 targeted protein-coding genes revealed that targeting TFs was significantly more frequent in HPr1AR (Supplementary Table 2C). Examples of cell-specific MRE are illustrated for the bookmarking factor *SMARCC1* and the CoA *TACC1* (Figure 3A).

Next, we tested how MRE and m6A-MRE related to changes in mRNA and protein expression, by exposing HPr1AR, LNCaP and 22Rv1 cells to an exogenous miR-96 mimic (50 nM, 48 h) and undertook RNA-Seq and LFQ proteomics; PCA plots are shown in Supplementary Figure 5. Reflecting the MRE distribution, the miR-96 dependent differentially expressed genes (DEGs) were largely distinct to each cell, with ~30% of HPr1AR and ~20% LNCaP DEGs being shared. Also, the miR-96 mimic impact is the effect of direct and indirect effects with <50% of all DEGs or differentially enriched proteins (DEPs) containing a MRE in the same cell background. For example, at the mRNA level (Figure 3B, **left**) in LNCaP there were ~470 MRE-containing DEGs, and in HPr1AR there were 260 MRE-containing (DEPs) (Figure 3B, **right**).

In all three cells, directly miR-96 bound targets were regulated both positively and negatively, although MRE containing protein-coding genes in 48h in HPr1AR and 24h in LNCaP were significantly more down-regulated than up-regulated (Figure 3C). In HPr1AR cells the m6A-MRE 48h genes were modestly less significantly down-regulated, whereas in LNCaP the miRNA-containing protein coding genes with m6A-MRE were significantly more down-regulated. This was also seen for proteins but only for m6A-MRE identified at 48h (Supplementary Figure 5B). This suggests that m6A-marked MRE have an increased likelihood to be associated with negative regulation.

Functional annotation with gene set enrichment analyses (GSEA) underscored the diversity of MRE-containing DEGs between HPr1AR and LNCaP. Analyses with the Hallmarks, Chemical and Genetic Perturbations and MiRNA libraries identified that the normalized enrichment scores (NES) were negative for all the 189 significant enrichment terms indicating that the downregulated DEGs were most significantly functionally grouped. The most significantly enriched terms (Supplementary Figure 5C) were different between HPr1AR and LNCaP, and whereas MYC-containing terms were shared between both cells (although most significant in HPr1AR), cell-cycle related terms such as related to E2F signaling were unique to LNCaP. Only one miRNA signature was enriched in either cell, and this was for miR-96 and only in LNCaP.

Across cell models annotation of all DEGs and DEPs to CoAs, CoRs, Mixed and TFs containing MRE or m6A-MRE, identified the most significantly regulated mRNA (Supplementary Table 3A) or protein (Supplementary Table 3B), positively or negatively, in either class. At the mRNA level, in HPr1AR the most significantly downregulated CoA with a MRE was the translation elongation factor, *EEF2*(87), and the most significant down-regulated CoR was *PDCD4*, which functions as a tumor suppresser(88). In LNCaP, *PDCD4* was also down-regulated, and the most negatively regulated of 14 miR-96 bound CoAs was *TACC1*. Similarly, the most significant TFs differed; in HPr1R it was the growth-responsive *ZFR36L1* whereas in LNCaP it was the AR-regulated *CREB2L2*. *RARG* was the most significantly regulated of three NRs (*AR*, *ESRRB* and *RARG*). At the protein level, the most significantly down-regulated CoA and CoR in both HPr1AR and LNCaP, was EEF1A1 and HSPA8 respectively. Interestingly, the NR, GR, was significantly upregulated in HPr1AR. Selecting for genes that were directly bound by miR96 in either HPr1AR or LNCaP, and negatively regulated by miR-96 mimics in HPr1AR, LNCaP and 22Rv1 cells revealed 54 genes. These genes included *RARG* and *TACC1*, as well as *ALDH9A1*, *DHCR24* and *CAPNS1* that are related to retinoid functions or signaling.

Finally, we tested how enrichment of proteins in the RARγ complex was targeted by miR-96 (Supplementary Figure 6, Supplementary Table 3C, 3D), and also tested whether the proteins enriched in the RARγ complex were both coregulators and contained MRE (Supplementary Table 3E), which further underscored that the RARγ complex is significantly enriched for coregulators that are bound by miR-96. For example, comparison of 22Rv1-RARγ and LNCaP RIME data revealed that differential enrichment of multiple MRE-containing genes (Figure 3D). These approaches demonstrated a significant role for miR-96 to target this complex and that MRE-containing genes were significantly enriched, most clearly in 22Rv1-RARγ-TACC1 cells and included the RNA-binding protein PRRC2C, as well as the DNA methyltransferase DNMT1 and NSUN2 (Supplementary Table 1A).

Together these data suggest that the cell background, exposure time (perhaps as a surrogate of miR-96 levels), and the presence of m6A-modifications all shape where and on which transcripts miR-96 binds to regulate RNA and protein expression. In general, the negative-regulated targets were more frequent, regulated to a greater magnitude and are enriched for functional networks. In LNCaP cells genes with m6A-MRE were most clearly negatively regulated supporting a role for this modification to enhance this function. Across three prostate cell models a consensus of directly bound and negatively regulated miR-96 targets included numerous retinoid-associated targets such as RARγ and TACC1 and were enriched in the RARγ complex.

### The RARγ complex augments AR enhancer interactions.

To test the genomic function of the RARγ-TACC1 complex we measured the coincidence of RARγ, AR and H3K27ac cistromes in 22Rv1 variant cells treated with either CD437 (400 nM) or DHT (10nM) for 6 h.

As anticipated, DHT treatment of 22Rv1-mock cells increased the AR cistrome from ~750 to ~1350 significant binding sites. Larger and more significant AR cistromes were identified in 22Rv1-RARγ and 22Rv1-RARγ-TACC1 cells (Supplementary Table 4A, Supplementary Figure 7A). Specifically, in 22Rv1-RARγ cells the DHT-dependent AR cistrome increased to ~5550, and in 22Rv1-RARγ-TACC1 cells, dramatically increased further to ~120,000 sites. Similarly, CD437 increased the significance of the RARγ cistrome in 22Rv1-RARγ. It is interesting to note, in passing, that the AR cistrome is extremely flexible not only over the choice of binding site, but also the number of sites. For example, a recent study on primary PCa revealed that across tumor samples the AR cistrome ranged from ~800 sites to more than 60,000 per sample, indicating considerable tailoring of the AR cistrome(89).

**Figure 4:**
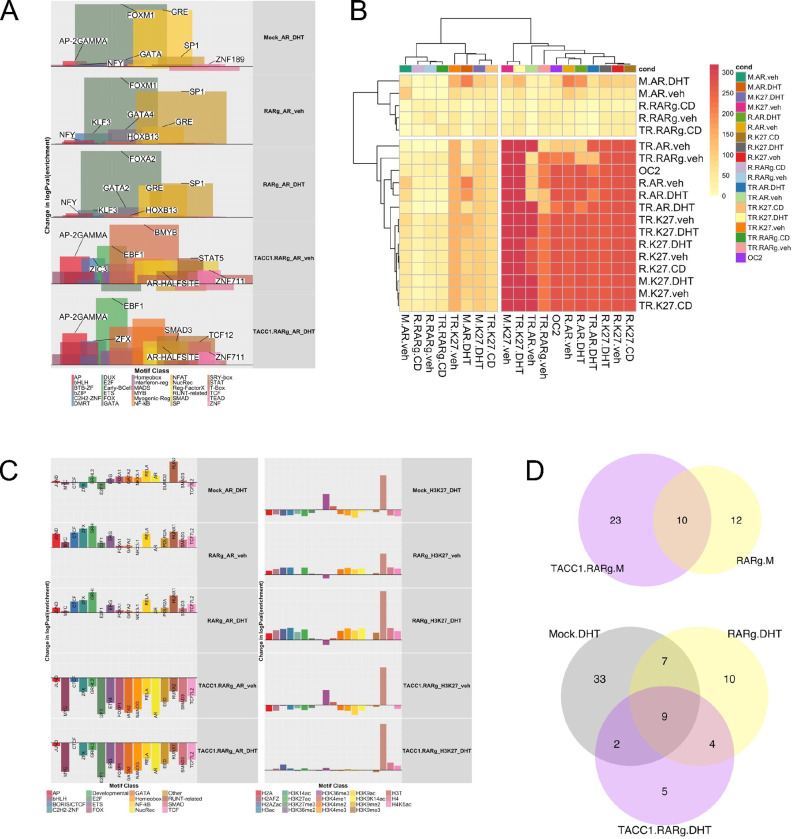
RARγ, AR and H3K27ac cistromes in 22Rv1 cell variants. 22Rv1-mock, 22Rv1-RARγ and 22Rv1-RARγ-TACC1 cells treated with DHT (10nM), CD437(400nM) or vehicle for 6h and CUT&RUN undertaken using antibodies to AR, RARγ, H3K27ac or IgG in triplicate. Differential enrichment of regions compared to IgG controls (p.adj < .1) was measured with csaw. **A**. Motif analyses for AR, RARγ, H3K27ac cistromes was undertaken (homer) and motifs were grouped into the indicated classes. For each motif the change in significance of enrichment was calculated across models compared to 22Rv1-mock cells treated with vehicle and per class the mean delta logPval enrichment and mean percentage coverage calculated (indicated by peak width), and the specific motif with the greatest delta per class is indicated. **B**. Cistromes were analyzed by GIGGLE and the change in enrichment calculated for transcription factors and cofactors (Left) and histone modifications (Right). The factor with the greatest change is labelled.

Motif analyses(90) also demonstrated how the RARγ-TACC1 complex impacted the AR cistrome (Figure 4A). The addition of DHT to 22Rv1-mock cells increased enrichment of motifs for the FOX family such as FOXM1, and NRs such as GR. In several aspects vehicle treated 22Rv1-RARγ cells recapitulated the effect of DHT on the AR cistrome in 22Rv1-mock cells, and also led to significant enrichment of SP1 motifs. Addition of DHT to 22Rv1-RARγ cells shifted the enrichment of the most enriched motifs within a class. For example, in basal and DHT-treated cells FOX motifs were positively enriched, but with DHT the most enriched member was FOXA2. By contrast, the addition of TACC1 reduced enrichment for NR and FOX motifs and increased the overall diversity of motif including AP, Early B-cell and MYB classes (Figure 4A; Supplementary Figure 7B). The motif enrichment in H3K27ac cistromes also reflected these patterns, in that the basal enrichment of H3K27ac in 22Rv1-RARγ cells was comparable to DHT treatment in 22Rv1-mock cells, and the addition of TACC1 increased the enrichment of ETS and ZNF motifs (Supplementary Figure 8A). Similarly, diversity of motif enrichment in the RARγ cistrome was increased by TACC1 (Supplementary Figure 8B).

We applied several strategies to test how RARγ expression impacted the levels and significance of the intersection of these 18 experimentally-derived cistromes. In the first instance we tested how the cistromes for each of RARγ, AR or H3K27ac overlapped in the different backgrounds, which revealed the shared and distinct sites; for example the basal AR cistrome in 22Rv1-RARγ cells had ~6500 sites not shared with other AR binding sites in other cells (Supplementary Figure 9). Given, we previously identified the ONECUT2 motif was enriched in RARγ binding sites (50), we also considered the 22Rv1 ONECUT2 cistrome (31) alongside these other cistromes.

To generate a more global understanding of how RARγ expression impacted AR, and H2K27ac cistromes, and their relationship with ONECUT2, we measured the significance of all binary cistrome interactions (n=361) and clustered the interactions by the -log10(p.adj) values (Figure 4B). This approach demonstrated that RARγ increased the significance of overlap of AR with H3K27ac cistromes; that is AR and H3K27ac were more significantly overlapped in 22Rv1-RARγ cells and even more so in 22Rv1-RARγ-TACC1 cells. Interestingly, compared to 22Rv1-Mock cells, AR in the presence of DHT was more significantly overlapped with ONECUT2 in 22Rv1-RARγ-TACC1 cells, but this was reduced in basal state. The significance of the overlaps of RARγ with AR was only modestly changing between cell backgrounds, suggesting that the biggest impact of RARγ was to facilitate the interaction of AR with H3K27ac. Similarly, by plotting the most significant overlaps in each cell background using either circos plots to illustrate their connectivity or bar charts to illustrate the change in significance also demonstrated that RARγ expression increased the frequency and level of the AR and H3K27ac cistrome overlaps, but this was not impacted by the RARγ-selective ligand CD437, suggesting this is a function of basal RARγ (Supplementary Figure 10A, B).

Next, we measured enrichment of these cistromes in either ChromHMM-derived epigenetic states(91) or publicly-available cistromes (76). RARγ alone, and with TACC1, significantly increased enrichment of the AR cistrome in ChromHMM-defined epigenetic states including at promoters and active enhancers. For example, the AR cistrome in DHT treated 22Rv1-Mock cells was enriched in promoters (logPV = 86.14), whereas in the 22Rv1-RARγ cells the enrichment was greater (logPV = 317.17); a similar RARγ-dependent enrichment occurred of AR at active enhancers (Supplementary Table 4B). Similarly, enumerating the frequency of peak to gene relationships from cistromes in each epigenetic state demonstrated that TACC1-RARγ expression increased the DHT-dependent frequency of AR associated gene relationships, notably in AR binding sites that overlapped with H3K27ac in active enhancers and promoters (Supplementary Figure 10C).

Comprehensive cistrome enrichment analyses with GIGGLE identified significant overlaps with multiple TF and histone modifications contained in the CistromeDB collection (> 10,000 total ChIP-seq datasets, across > 1100 factors) (Figure 4C). In the first instance, TFs were grouped into broadly similar classes as used for motif analyses, and again supported the concept that expression levels of RARγ recapitulated the action of DHT in 22Rv1-mock. For example, DHT treatment in 22Rv1-mock cells increased the significance of the overlap of AR with various classes of TF including ETS (e.g. ERG), FOX (e.g. FOXA1), GATA (e.g. GATA2), NF-kB (e.g. RELA) and RUNT-related (e.g. RUNX1). Reflecting the motif enrichment findings, this was broadly similar in vehicle-treated 22Rv1-RARγ cells, which also included gains of enrichment in ZNF factors, developmental factors, and gain of SMAD and TCF factors. The addition of TACC1 did not increase the significance of shared binding but this may reflect the larger size of the RARγ-TACC1 dependent AR cistromes. Enrichment of histone modifications within the H3K27ac cistrome also suggested that the RARγ expression phenocopied DHT-treatment in 22Rv1-mock cells, and that TACC1 expression facilitated shared enrichment of H3K27ac with histone modifications that are hallmarks of active transcription such as H3K36me3. H3K27ac cistromes also demonstrated a selective role for RARγ-TACC1 to lead to overlap with ETS, FOX and ZNF TFs (Supplementary Figure 11).

The H3K27ac cistromes were used to define the distribution of super enhancers (SE) using the ROSE algorithm, and specifically used BRD4 from 22Rv1 cells(92,93) as a pilot SE-enriched factor to generate high confidence sites. Interestingly, under these stringent conditions, there were no SE identified in Mock-22Rv1 cells treated with vehicle, whereas SE were identified in 22Rv1-RARγ and 22Rv1-RARγ-TACC1 cells in both the vehicle and DHT-treated conditions (Figure 4D). Annotation of the SE revealed ~600 genes associated across all vehicle-treated cells, and ~525 in the DHT-treated cells. Annotation of these genes as to whether they were coregulators or TFs also supported a unique RARγ-mediated SE-dependent function to regulate NR were annotated to these SE, including NR4A1/NUR77 and THRB (Supplementary Table 4C).

Together these cistrome data support the concept that restored RARγ expression alone phenocopied the impact of DHT treatment in 22Rv1-Mock cells by increasing the number of AR binding sites, the repertoire of enriched motifs, the overlap with ONECUT2, and also shared binding with publicly available cistrome data sets. RARγ expression also enriched the AR cistrome in active enhancers and promoters and the presence of TACC1 increased the frequency of DHT-dependent peak:gene relationships and also enriched for unique SE sites.

### The RARγ complex is has shared binding with bookmarking factors and promotes DHT-dependent AR cistrome in mitotic cells.

The RARγ complex contains bookmarking factors, and RARγ expression shaped the AR cistrome, and alone recapitulated many actions of the addition of DHT in terms of the transcriptome, motif enrichment, overlap with other cistromes and even the ability to activate SE. We therefore tested the possibility that RARγ exerts this impact on AR function because of a mitotic bookmarking function. To test these possibilities, we examined if RARγ expression impacted accessible chromatin, shared binding with bookmarking factors and measured its impact on the AR cistrome during mitosis.

**Figure 5:**
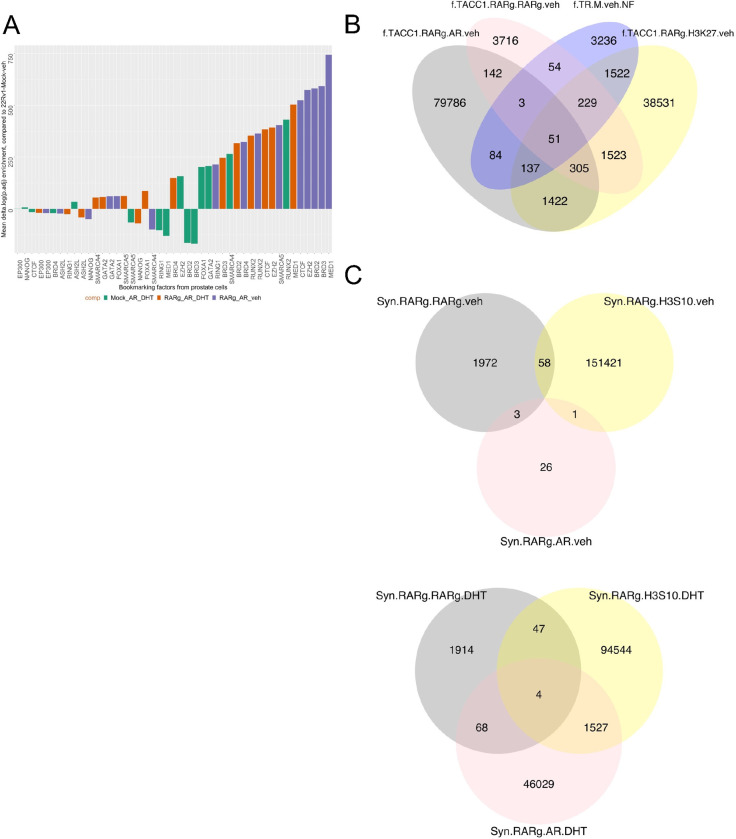
The role of RARγ in enhancer bookmarking. **A.** The shared binding of AR cistromes in 22Rv1 cell variants with bookmarking factors. **B.** ATAC-Seq was undertaken in triplicate in 22Rv1 cell variants in vehicle treated controls control. FASTQ files were QC processed, aligned to hg38 (Rsubread), before further processing with ATACseqQC to define nucleosome free regions. Differential enrichment of regions was measured with csaw and the significantly different regions (p.adj < .1) were then intersected with the indicated AR and RARγ cistromes to generate the Venn diagrams of overlapping regions by a minimum of 1bp (ChIPpeakAnno). **C**. 22Rv1 cell variants were treated with nocodozole (60 ng, 18h) or vehicle control and then treated with DHT (10nM) or vehicle for 6h and CUT&RUN undertaken using antibodies to AR, RARγ, H3S10P or IgG in triplicate. Differential enrichment of regions compared to IgG controls (p.adj < .1) was measured with csaw and Venn diagrams generated of overlapping regions by a minimum of 1bp (ChIPpeakAnno).

There was a significant overlap of the RARγ-dependent AR cistrome with bookmark factor cistromes (Figure 5A), and therefore we undertook ATAC-Seq to identify RARγ-dependent nucleosome-free (NF) regions across the 22Rv1 variants cells, that overlapped with H3K27ac, and were bound by both AR and RARγ. Specifically, we identified NF regions that were gained in 22Rv1-RARγ-TACC1 cells compared to 22Rv1-mock counterparts, and therefore were RARγ-TACC1 dependent and overlapped these with basal AR and H3K27 cistromes. This revealed that ~1950 NF regions that overlapped with H3K27, and 280 directly shared with RARγ and 188 with AR, which suggests that RARγ directly and indirectly impacts accessible chromatin as there ~1500 sites that are NF and H3K27ac in a RARγ dependent manner but not RARγ bound, although we cannot exclude that these sites had been previously marked by RARγ (Figure 5B).

We synchronized 22Rv1-RARγ cells with nocodozole (60 ng, 18h) or left untreated (asynchronous), and undertook CUT&RUN for AR, RARγ and H3S10P, as a marker of mitotic cells. This treatment resulted in ~70% of cells in G_2_/M. In asynchronous cells the number of AR and RARγ peaks were broadly comparable to the earlier experiments, for example with ~6000 basal AR and ~400 basal RARγ sites (Supplementary Table 6). In synchronized cells the number of RARγ peaks increased to ~2000 and was consistent between vehicle and DHT treated cells (Figure 5C). The number of AR peaks in vehicle-treated synchronized cells was very modest but was strikingly increased to ~46,000 by DHT.

Together these data suggest that the RARγ complex, which contains bookmarking factors displays shared binding with bookmarking factors, that RARγ-TACC1 exerts a profound effect on NF regions that are marked by H3K27ac, and that RARγ binding is increased in G_2_/M cells and profoundly increased the magnitude of the DHT-dependent AR cistrome.

### Restoration of the RARγ-TACC1 complex redirects the AR-dependent transcriptome and enhances enzalutamide sensitivity.

Phenotypically, 22Rv1-RARγ cells grew more slowly than 22Rv1-Mock cells, with more cells in G_1_ phase of the cell cycle. To test the transcriptomic impact of the RARγ-TACC1 complex we used RNA-Seq in the 22Rv1 variants treated for 24h with DHT (10nM), ENZA (10μM), or CD437 (400nM).

**Figure 6:**
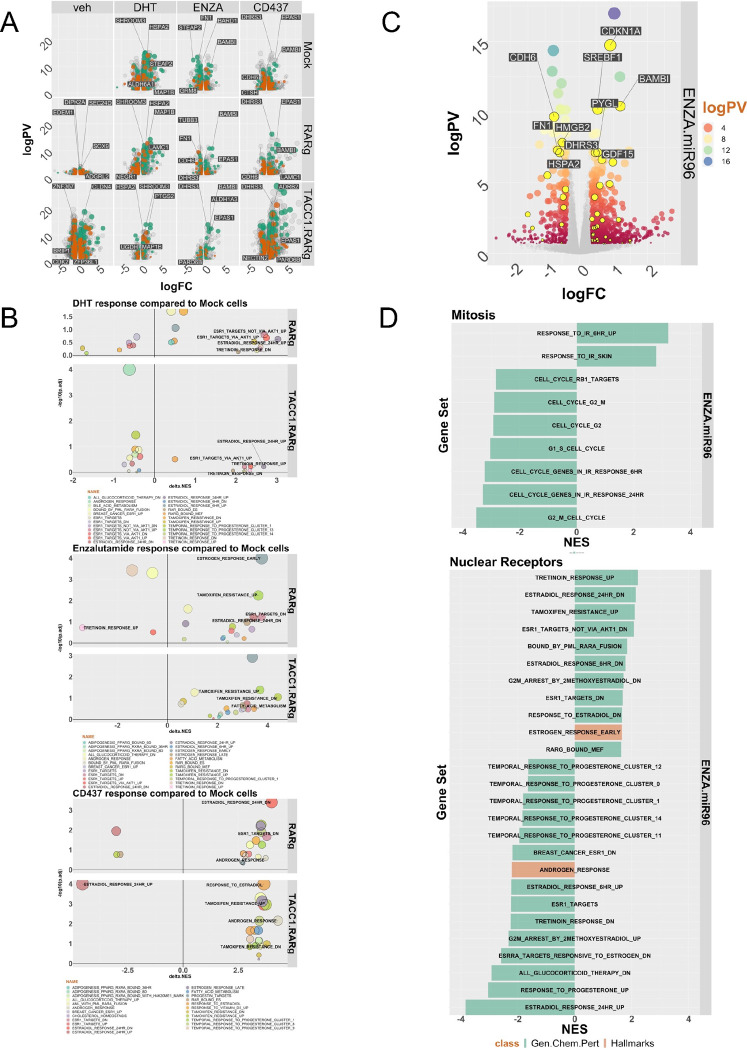
The RARγ-dependent transcriptome in response to DHT, Enza or CD437 in 22Rv1 cell variants. 22Rv1 cell variants were treated with DHT (10nM), CD437 (400nM) or ENZA (10μM) for 24h in triplicate and RNA-Seq undertaken. FASTQ files were QC processed, aligned to hg38 (Rsubread), and differentially expressed genes (DEGs) ) (logPV > 1 & absFC > .37) identified with edgeR. **A.** Volcano plot depicting DEGs in 22Rv1 cell variants and treatments, with luminal (teal) and basal (brown) genes indicated. **B**. Pre-ranked gene set enrichment analysis (GSEA) was undertaken using the Hallmarks, and Chemical and Genetic Perturbations terms, and the most frequently enriched terms filtered (e.g. NRs, epigenetics and cell cycle terms). The delta normalized enrichment score (NES) between terms in either 22Rv1-RARγ or 22Rv1-RARγ-TACC1 cells compared to 22Rv1-mock cells are illustrated. **C**. Volcano plot depicting DEGs in 22Rv1 cells treated with Enza (10μM), miR-96 antagomir (50nM) or the combination. MiR-96 bound and regulated genes are indicated (yellow). **D**. GSEA analyses undertaken as **B**. and the most enriched terms in NRs or mitosis indicated in order of NES.

Principal component analyses revealed that experimental conditions explained most of the variation in expression (Supplementary Figure 12A), and volcano plots (Figure 6A) illustrate the DEGs for each cell background either in the basal state or following the treatments. The largest change was the basal 22Rv1-RARγ-TACC1 cells compared to 22Rv1-mock (~5500 DEGs) and smallest was 22Rv1-RARγ-TACC1 cells treated with Enza (~500 DEGs). We classified these DEGs with several enrichment approaches; luminal and basal prostate gene sets; coregulators; NRs; intronic-encoded miRNA, miR-96 bound and differentially regulated by miR-96 mimics (Supplementary Table 6A).

Luminal and basal (94) target signatures include both up- and down-regulated genes, and therefore we investigated whether these signatures were altered in 22Rv1 variant DEGs; for example 22Rv1-RARγ-TACC1 cells displayed the most frequent changes in luminal genes in responses to CD437, and to a lesser extent with Enza. (Supplementary Figure 12B). Classification of coregulators revealed that distinct cohorts were regulated by DHT such as the *FZD5* and *FZD1* in 22Rv1-RARγ cells, and *TIMELESS* was downregulated in 22Rv1-RARγ-TACC1 cells. Restricting the analyses to NRs revealed several cell backgrounds and treatment combinations where the frequency of NRs was significantly enriched (Supplementary Table 6B), for example in CD437 treated 22Rv1-RARγ cells, and Enza treated 22Rv1-RARγ-TACC1 cells the altered NRs included Type II and orphan members *NR2A1/HNF4A*, *NR2F2*, *NR2F1*, and *ESRRG*. Similarly, miR-96 targeted and regulated genes (n~660) were significantly enriched, but only in the DEGs from 22Rv1-RARγ and 22Rv1-RARγ-TACC1 cells (Supplementary Table 6C).

To reveal broader enrichment patterns in the DEGs, GSEA was undertaken using the Hallmarks, and Chemical and Genetic Perturbations terms, and the most frequently enriched words in the terms were ranked. This revealed that terms related to either NRs, epigenetics and cell cycle were most common; Supplementary Figure 12C is a waterfall plot of the most enriched NR terms across 22Rv1 variants. We calculated the delta between NES for the same terms in either the 22Rv1-RARγ or 22Rv1-RARγ-TACC1 cells compared to 22Rv1-mock cells (Figure 6B). This revealed that DHT treatment in 22Rv1-RARγ and 22Rv1-RARγ-TACC1 cells led to commonly and positively enriched estrogen-associated terms, and also in 22Rv1-RARγ-TACC1 genes regulated by the synthetic retinoid, Tretinoin. By contrast, Enza treatment positively enriched for tamoxifen-associated terms and negatively enriched for Tretinoin-dependent genes. CD437 also enriched for estrogen-dependent terms, but also positively increased the enrichment of androgen-dependent terms.

Because the RIME analyses revealed enrichment of splice factors in the RARγ complex, we also measured alternatively spliced RNA expression (Differentially Expressed Transcripts, DETs). For 22Rv1-RARγ cells compared to 22Rv1-mock (Supplementary Table 6D), the DETs were again categorized as either coregulators (e.g. CoA or CoR), containing a MRE or intragenic-miRNA. Although the total number of DETs was broadly comparable across cells and treatments, the significance and the class enrichment varied. For example, in Enza treatment of 22Rv1-RARγ cells compared to 22Rv1-mock the significant changes included the mitotic spindle regulator, SPAG5, and the AR itself, which was detected at the protein level with reduced expression of ARv7 (Supplementary Figure 13A, B).

Given that expression of the RARγ complex significantly shaped the response to Enza, and that miR-96 targets are significantly enriched in the RARγ complex, we next tested how miR-96 antagomirs could be used to sensitize the 22Rv1 cells to Enza. That is, we reasoned miR-96 antagomirs could be used to depress RARγ and restore AR functions.

At the phenotypic level we examined viability, clonogenicity, programmed cell death and cell cycle profiles. The miR-96 antagomir (50nM) plus ENZA (10μM) significantly decreased cell viability (Supplementary Figure 14A), which was associated with a modest reduction of S-phase cells and an increase in necrotic cells at 48h. At the transcriptomic level, the miR-96 antagomir and ENZA co-treatment resulted ~1100 DEGs compared to individual treatments including ~50 MRE-containing miR-96 regulated genes (Supplementary Figure 14B, Figure 6C (yellow)). GSEA analyses also revealed enrichment of Estrogen and Tretoinin treatment terms reflecting the impact of RARγ expression (Figure 6B), as well as genes regulated by the leukemic chimeric protein PML-RARA and bound by RARγ (Figure 6D). Likewise, the DETs in 22Rv1 cells treated with miR-96 antagomir plus Enza (Supplementary Table 7A; Supplementary Figure 15). were enriched for CoAs, notably including three SMARC genes that participate in the BAF complex that are regulate nucleosome positioning (Supplementary Table 7B).

Together these transcriptomic data demonstrate a significant role for the RARγ complex to impact the AR dependent transcriptional functions, including to promote luminal differentiation gene programs and to alter the responses to Enza. Also underscoring the interdependence of RARγ and miR-96, MRE genes were significantly enriched in the RARγ-dependent transcriptomes, and a miR-96 antagomir can enhance Enza responses in 22Rv1 cells.

### The RARγ complex augments AR cistrome-transcriptome relationships and antagonizes ONECUT2-regulated transcriptomes.

We sought to test the significance of the RARγ-dependent AR cistromic and AR transcriptomic relationships. In each cell background (22Rv1-mock, 22Rv1-RARγ and 22Rv1-RARγ-TACC1) we tested the relationships the ChromHMM-classified cistrome gene:peak relationships with the DHT and Enza-regulated genes through several approaches. Firstly, we calculated the frequency of peak:gene relationships in 10kb bins around DEGs, which revealed that the AR and H3K27ac binding sites annotated to Enza-regulated genes only in 22Rv1-RARγ cells but not 22Rv1-mock cells (Supplementary Figure 16A).

**Figure 7.**
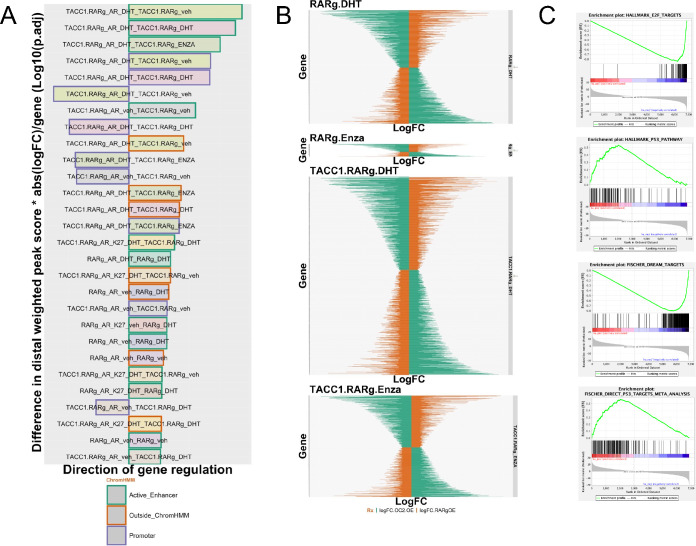
RARγ-dependent AR cistrome-transcriptome relationships. **A.** each cell type/target cistrome was overlapped with ChromHMM defined epigenetic states annotated to genes within 100 kb that was also a DEG. The weighted distal peak significance was summed for each gene and multiplied by the absolute fold change for the same DEG. This score is the weighted cistrome-transcriptome (wt-C-T). A Wilcox test was used to compare the significant differences between the wt-C-T values to the comparable values in 22Rv1-mock cells. This revealed that the most significantly impacted cistrome-transcriptome relationships were TACC1-RARg-dependent. The name of bar refers to the cistrome first (e.g. TACC1_RARg_AR_DHT) and then the transcriptome it is matched to (e.g. TACC1_RARg_CD437). The direction of the bar refers to whether the genes were up or downregulated. The order refers to the most significance of change between the wt-C-T and that in 22Rv1-Mock cells. **B.** Antagonistic gene expression between either DHT or Enza regulated genes in 22Rv1-RARγ cells and 22Rv1-RARγ-TACC1 cells compared to gene regulation by over-expression of ONECUT2. Transcriptomic data in 22Rv1-RARγ cells to 22Rv1 cells with ONECUT2 over-expression, and defined gene expression as cooperative, if for a given gene the direction of change was the same, and antagonistic if the direction of change was opposite. The height of each panel is proportional to the number of events. **C**. GSEA identified negative enrichment of E2F genes and positive enrichment of p53 genes in the Enza-regulated antagonized genes in 22Rv1-RARg-TACC1 cells.

Secondly, we tested if RARγ-dependent augmentation of ChromHMM-categorized AR cistrome significantly impacted the dependent-transcriptome relationships(21) by applying a modified BETA method as previously(83,95). We constructed a weighted cistrome-transcriptome score per peak binding site per gene per cell background (wt-C-T), and tested whether these values were significantly changed between ChromHMM states, treatments and cell backgrounds; all relationships are shown in Supplementary Figure 16B. The top 30 most significantly changed wt-C-T relationships are shown in decreasing order in Figure 7A and revealed that RARγ-TACC1 prominently increased the strength of the relationships between AR binding in Active Enhancers or Promoters, and to a lesser extent in Poised Enhancers, and the regulation of genes at the basal level and in response to both DHT and Enza. Also these relationships were more common for upregulated genes, they did include downregulated genes. Therefore RARγ-TACC1 not only augments AR binding but also enhances its gene-regulatory capacity.

Thirdly, we examined if RARγ impacted how AR and ONECUT2 governed gene expression. We reasoned that RARγ-TACC1 may antagonize ONECUT2 and impact how cells responded to Enza. Therefore, we compared the impact of RARγ-TACC1 and ONECUT2 expression on the transcriptomic responses of DHT and Enza responses in 22Rv1 cells (31). We examined whether the genes regulated by DHT or Enza genes were enriched for cooperative or antagonistic gene targets of RARγ and ONECUT2. We defined gene expression as cooperative if the direction of change for a given gene was the same from both RARγ and ONECUT2 restored expression, and antagonistic if the direction of change was opposite.

In 22Rv1-RARγ cells there were ~750 genes that were DHT-regulated genes and antagonized by RARγ and ONECUT2, but only ~150 Enza-regulated genes were antagonized. Addition of TACC1 increased the antagonized DHT-regulated genes to ~1200 and Enza-regulated genes to ~700 (Figure 7B). This suggests that TACC1-RARγ can function in response to Enza to antagonize genes regulated by ONECUT2. By contrast the impact on expression of cooperative genes was approximately equal and not impacted by TACC1 (Supplementary Figure 17A). GSEA analyses of the Enza-regulated genes in 22Rv1-RARγ-TACC1 cells that antagonized the effect of ONECUT2 revealed positive enrichment for p53 targets, Estrogen signaling and negative enrichment for E2F targets (Figure 7C). Genes that were antagonized between RARγ and ONECUT2 were significantly associated (logPV = 22.5) with unique AR binding sites, for example at the ONECUT2 locus.

### RARγ-centric gene signatures are significantly enriched in metastatic PCa.

RARγ and ONECUT2 cistromes significantly overlap (Supplementary Figure 8), RARγ-TACC1 reduced the shared binding of the basal AR and ONECUT2 (Figure 4B), and there is an intricate interplay between RARγ, AR, and ONECUT2. Therefore we tested if the miR-96 bound and regulated genes (termed miR96 BR; n~660), and the RARγ-TACC1 and ONECUT2 antagonized genes (termed RARγvsOC2; n~1400) maybe associated with PCa progression, metastasis and be permissive of alternative lineages, for example as displayed by the Pten^−/−^ Rb^−/−^ genetically engineered mouse model of PCa (30).

**Figure 8.**
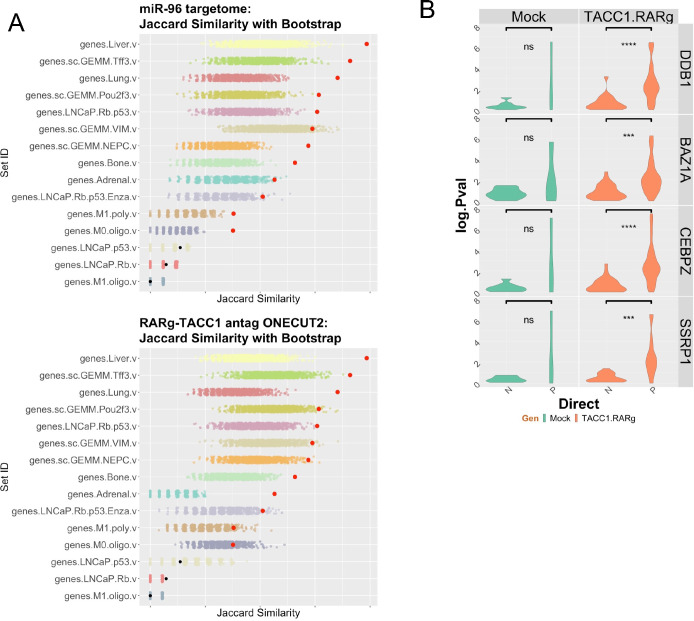
Clinically significant relationships between RARγ and advanced PCa. A. Two gene lists were generated; the miR-96 targetome of 663 MRE bound and miR-96 regulated genes, and the 704 RARγ-ONECUT2 antagonized genes. Specifically, the miR-96 targetome genes were extracted from LNCaP cells to generate a ranked list, and similarly the RARγ-ONECUT2 antagonized genes were extracted from 22Rv1 cells to also generate a ranked list. The similarirt of these rank genes was compared to the indicated ranked gene lists from the indicated experiments and similarity measured by Jaccard similarity measurements, and the significance determined by bootstrapping (n=1000). The symbol in red are significant similarity. **B.** Partial correlation analyses in in SU2C tumors (n=293) between AR and RARγ-dependent AR-H3K27ac DHT regulated genes considering the impact of the indicated coregulators. The change in the correlation (delta.corr) was calculated as the difference between the Pearson correlation and Pearson partial correlations between AR and these target genes and each of the indicated coregulators.

To test this possibility, we applied several approaches. In the first instance we tested how the miR96 BR and RARγvsOC2 gene lists were captured in publicly available gene sets, including the Pten^−/−^Rb^−/−^ GEMM; LNCaP cells with p53 and RB knockdown(96); metastatic PCa(97). Specifically we used comparison of ranked order of genes between sets using Jaccard similarity analyses with bootstrapping. This revealed that both miR-96 regulated genes, and RARγ and ONECUT2 antagonized genes, were enriched in different metastatic sites, notably liver. The ranked gene lists were also enriched in both murine and human models with combined disruption of p53 and RB, including selectively enriched in different lineages, most significantly in the Tff3 lineage (Figure 8A).

To examine how components of the RARγ complex impacted AR gene regulation we used partial correlation analyses to test the impact of coregulators in the RARγ complex on the relationship between AR expression and genes associated with AR bound in active enhancers by using the SU2C cohort of advanced PCa (Figure 8B). This demonstrated that several of the coregulators in the RARγ complex, including SSRPI(98) and BAZ1A(95), significantly and positively impacted the strength of the correlations between AR expression and AR enhancer dependent target genes.

Finally, we also examined relationships between RARγ, TACC1 and ONECUT2 in the SU2C cohort. *RARG* expression positively correlated with *TACC1* (Spearman’s correlation, .34; qval 2.6e^−6^) and negatively correlated, albeit modestly, with *ONECUT2* (−.2; qval=0.02). We therefore separated tumors in upper quartile *RARG* and lower quartile *ONECUT2*, and *vice versa*, and identified DEGs between these tumor groups (n~25 tumors each group). The top DEGs significantly separated tumors by AR score (X^2^=50.9; pval = 9.6e^−13^) and whether a patient had been exposed to ARSI (X^2^=4.3; p-value = 0.03) (Supplementary Figure 17B). Together, these findings suggest that RARγ-TACC1 and ONECUT2 are functionally intertwined and through shared binding and co-regulation of a significant number of target genes that promote sustain AR signaling and the response to Enza.

## DISCUSSION

The current study aimed to investigate how NRs may crosstalk to control cell lineage decisions by examining the impact of nuclear resident Type II NRs on the actions of Type I NRs with the goal to establish possible bookmarking like functions. We focused on the RARγ complex and its regulation by miR-96, an oncomir(50,56,99–101), and demonstrated this receptor can regulate AR signaling in a clinically significant manner through a bookmarking mechanism.

We generated a series of high-dimensional data sets to understand how RARγ complex, its upstream regulation and its downstream signaling within and across different PCa models. The RARγ complex included CoAs, CoRs, mixed function coregulators, TFs and RBPs, and functionally the complex was enriched in processes related to chromatin accessibility but also RNA processing and splicing, as well as bookmarking factors. Recently(40), roles for BAF components to bookmark have demonstrated that SMARCB1 and SMARCA4 displayed mitotic binding sites in mitotic ES cells, although was most pronounced for SMARCB1, and these were enriched in the RARγ complex.

The RARγ complex was significantly targeted by miR-96, and by examining the transcriptome and proteome, and the impact of the RNA modification m6A we were able to reveal some subtleties for m6A-MRE, for example selectively negatively regulating miRNA-containing genes in LNCaP cells and impacting the distribution of miRNA across protein-coding but not non-coding genes. This contributes some explanation as to why the miR-96 targetome is time, cell and RNA-species specific, and the impact of m6A readers and writers appear impactful. More specifically, we identified m6A regulators such as YTHDC1, RBM15, and METTL3, in the RARγ complex in 22Rv1-RARγ cells.

Next, we measured how RARγ levels impacted AR cistromes in three isogenic cell backgrounds, namely 22Rv1-Mock, 22Rv1-RARγ and 22Rv1-RARγ-TACC1 cells. Whereas the RARγ cistrome overlaps were essentially unchanging in the different cell backgrounds, RARγ significantly enhanced AR binding in active enhancers and significantly changed the repertoire of super-enhancers. Furthermore RARγ and TACC1 together reduced the significance of the overlap of ONECUT2 and AR. In many aspects, the function of RARγ on the AR appeared to phenocopy the effect of DHT exposure as demonstrated with motif enrichment and comprehensive cistrome overlaps. One aspect of this was associated with RARγ complex being enriched for bookmarking factors, and the RARγ-dependent AR cistrome significantly overlapping with prostate-specific bookmark cistromes. Indeed, cells in G_2_/M were acutely sensitive to RARγ expression which massively increased the DHT-dependent AR cistrome.

Reprogrammed AR sites to sentinel sites in ARSI-resistant PCa are prepopulated by other TFs rather than created as de-novo(2). The current data supports a model whereby RARγ enhanced and redirected AR enhancer usage, which was further amplified in presence of TACC1. The robust convergence of RARγ functions in guiding AR signaling was observed through the binding of transcription regulatory elements and interactions among nuclear proteins. These gained AR sites were enriched for GATA and Homeobox motifs, and significantly overlapped with H3K27ac and super-enhancers. Thus, these data support a model whereby RARγ promotes and enhances AR functions to sustain DHT-dependent luminal differentiation programs, which also are important for Enza sensitivity. Indeed, this raised the intriguing possibility of restoring Enza functions and to augment ARSI-centered therapies with miR-96 antagomir. Treatment of 22Rv1 cells with Enza changed the transcriptome significantly and also altered the splice variants and notably reduced expression of the AR variant, AR-V7.

Set against this, ONECUT2 significantly overlaps with RARγ and AR cistromes, but appears to have an opposing function to promote alternative lineages. These data raise the question as to how antagonism may arise at shared RARγ and ONECUT2 enhancers. One explanation is due to low density CpG methylation at enhancer regions which can both repress and attract different classes of TFs. Specifically, high throughput approaches have revealed that CpG methylation status of ~ 500 motifs differentially impact TF binding; ~40% showed decreased binding with methylation and ~60% showing increased binding(102). Of relevance here, increased CpG methylation attracts RARγ binding and repels ONECUT2(102); therefore, the status of CpG at enhancer sites will impact the functional antagonism between RARγ and ONECUT2. It is worth noting that RIME revealed that the RARγ complex in 22Rv1-RARγ-TACC1 cells was enriched for the DNA methylatransferase DNMT1. These findings are in keeping with the concept that changes in CpG methylation levels at enhancer regions are highly dynamic, critical in development(103,104) and appear to promote and sustain cell differentiation(105–112).

In summary the current study supports a model whereby the RARγ complex exerts a bookmarking function for AR to shape cistromic functions, and in turn is regulated by miR-96. Androgen-indifferent lineages evoked by lineage plasticity drivers, such as ONECUT2, may be prevented by combinatorial strategies that induce RARγ and maintain RARγ complex bookmarking factors in favor of luminal differentiation programs at competing regulatory regions.

## Supplementary Material

Supplement 1

Supplement 2

Supplement 3

## Data Availability

The datasets generated and/or analyzed during the current study will be available on GEO
