# Supplementary material for: Epigenetic disruption of the RARγ complex impairs its function to bookmark AR enhancer interactions required for enzalutamide sensitivity in prostate cancer
Source: bioRxiv. 2024 Feb 5:2023.12.15.571947. Preprint. [Version 3] doi: 10.1101/2023.12.15.571947 (PMC10760102; doi:10.1101/2023.12.15.571947)
Supplement: Supplement 3 [file media-3.pdf]

Epigenetic disruption of the RARγ complex impairs its function to bookmark AR enhancer interactions required for enzalutamide sensitivity in prostate cancer.

Sajad A Wani[1a], Shahid Hussain[2,3*], Jaimie S Gray[1b*], Debasis Nayak[1c], Hancong Tang[1e], Lillian M Perez[4], Mark D Long[5], Manjunath Siddapa[1f], Christopher J McCabe[6], Lara E Sucheston-Campbell[7], Michael R Freeman[4], Moray J Campbell[2,3*]

[1]Division of Pharmaceutics and Pharmacology, The Ohio State University, Columbus, OH 43210
[2]Division of Cancer Biology, Cedars Sinai Cancer, and [3]Board of Governors Innovation Center, Department of Biomedical Sciences, Cedars-Sinai Medical Center, Los Angeles, CA 90048,
[4]Division of Cancer Therapeutics, Cedars Sinai Cancer, Departments of Urology and Biomedical Biomedical Sciences, Cedars-Sinai Medical Center, Los Angeles, CA 90048
[5]Roswell Park Comprehensive Cancer Center, Elm and Carlton Streets, Buffalo, NY 14263
[6]Institute of Metabolism and Systems Research (IMSR), and Centre of Endocrinology, Diabetes and Metabolism (CEDAM), University of Birmingham, Birmingham, UK
[7]Department of Cancer Prevention and Control, Cedars-Sinai Medical Center, Los Angeles, CA 90048

Supplementary tables
Supplementary figure legends

Cell.Rx	Direct	Coreg	Overlap	logPV.hyper	MostSignificant
22RV1.RARg.TACC1_veh	P	Mixed	272	290.68	HCFC1
LNCaP_veh	P	CoA	216	256.71	NSUN2
22RV1.RARg_CD437	P	TF	384	247.90	RARG
22RV1.RARg_CD437	P	CoR	232	234.59	PA2G4
22RV1.RARg_CD437	P	Mixed	200	197.66	HCFC1
22RV1.RARg_veh	P	CoR	160	168.17	DNMT1
LNCaP_CD437	P	CoA	128	130.32	NSUN2
22RV1.RARg_veh	P	TF	192	92.86	RARG
22RV1.RARg_veh	P	Mixed	104	89.14	HCFC1
LNCaP_veh	P	CoR	96	76.21	NCOR2
LNCaP_CD437	P	TF	128	63.81	RARG
LNCaP_veh	P	TF	152	60.50	RARG
LNCaP_veh	P	Mixed	72	51.19	RBM15
LNCaP_CD437	P	CoR	48	29.67	PA2G4
LNCaP_CD437	P	Mixed	40	23.89	RBM15

Supplementary Table 1A: Enrichment of coregulators in the RARγ complex. LNCaP, 22Rv1, 22Rv1-RARγ and 22Rv1-RARγ-TACC1 cells in quadruplicate were treated with vehicle or CD437 (400 nM, 6 h), and RIME undertaken to identify significantly and positively enriched proteins in the complex, which were then annotated as to whether they were either a coactivator (CoA), corepressor (CoR), mixed function coregulator (mixed) or a transcription factor (TF). A hypergeometric test was undertaken to examine if the overlaps for each group of proteins were significantly enriched in the RARγ complex, and the most positively enriched protein is indicated.

Cell.Rx	Direct	MostSignificant
LNCaP.CD437 vs veh	P	RXRA
LNCaP.CD437 vs veh	P	ESRRA
LNCaP.CD437 vs veh	P	HNF4G
LNCaP.CD437 vs veh	P	NR2F2
LNCaP.CD437 vs veh	P	NR2C1
LNCaP.CD437 vs veh	P	NR2C2
LNCaP.CD437 vs veh	P	AR
LNCaP.CD437 vs veh	P	RXRB
RV1 vs LNCaP RARg.veh	P	RXRB
RV1 vs LNCaP RARg.veh	P	RARG
RV1 vs LNCaP RARg.veh	P	RXRA
RV1 vs LNCaP RARg.veh	P	NR2C1
RV1 vs LNCaP RARg.veh	P	NR2C2
RV1 vs LNCaP RARg.veh	P	AR

Supplementary Table 1B: Change in enrichment in nuclear receptor targets in the RARγ complex. LNCaP, 22Rv1, 22Rv1-RARγ and 22Rv1-RARγ-TACC1 cells in quadruplicate were treated with vehicle or CD437 (400 nM, 6 h) and RIME undertaken. In each case the change in enrichment was then calculated between the indicated conditions to identify differentially enriched proteins, both negatively and positively enriched in the complex, which were then annotated as to whether Nuclear Receptors. A hypergeometric test was undertaken to examine if the overlaps for each group of proteins were significantly enriched in these altered enrichments in the RARγ complex, and the most significantly altered protein is indicated.

Cell.Rx	Overlap	logPV.hyper	Threshold.hyper	MostSignificant
22RV1.RARg.TACC1_veh	44	4.50	Significant	SMCHD1
22RV1.RARg.TACC1_CD437	45	4.16	Significant	TRRAP
22RV1.RARg_veh	20	2.07	Significant	SMCHD1
LNCaP_CD437	14	1.85	Significant	PLEC
22RV1.RARg_CD437	28	1.81	Significant	SMCHD1

Supplementary Table 1C: Enrichment of coregulators in the RARγ complex. LNCaP, 22Rv1, 22Rv1-RARγ and 22Rv1-RARγ-TACC1 cells in quadruplicate were treated with vehicle or CD437 (400 nM, 6 h), and RIME undertaken to identify significantly and positively enriched proteins in the complex, which were then annotated as to whether they were in the list of mutated chromatin remodelers; so-called long-tail mutants. A hypergeometric test was undertaken to examine if the overlaps for each group of proteins were significantly enriched in the RARγ complex, and the most positively enriched protein is indicated.

Cell	Exper	Time	n
HPr1AR	IMPACT	24h	472
HPr1AR	IMPACT	48h	7494
HPr1AR	m6A.IMPACT	24h	241
HPr1AR	m6A.IMPACT	48h	7258
LNCaP	IMPACT	24h	3224
LNCaP	IMPACT	48h	333
LNCaP	m6A.IMPACT	24h	2888
LNCaP	m6A.IMPACT	48h	263

Supplementary Table 2A. Frequency of miR-96 and m6A-miR-96 binding sites by time and cell background. MiR-96 binding sites were identified by IMPACT-Seq in HPr1AR and LNCaP cells by transfecting cells with a biotinylated miR-96 mimic (50 nM) and after 24 and 48 h cells were lysed and miR-96 interacting mRNA isolated by streptavidin beads, eluted, sequenced, and aligned to the transcriptome and discrete interacting sites (IMPACT) identified by `csaw` (n). In parallel, m6A-Seq was undertaken in the same cells and the overlap of significantly shared sites with m6A at a miR-96 binding sites (m6A.IMPACT) with `bedtools` and the frequency also enumerated (n).

Cell	Exper	Time	n
HPr1AR	IMPACT.genes	24h	423
HPr1AR	IMPACT.genes	48h	2859
HPr1AR	m6A.IMPACT.genes	24h	227
HPr1AR	m6A.IMPACT.genes	48h	2859
LNCaP	IMPACT.genes	24h	2381
LNCaP	IMPACT.genes	48h	245
LNCaP	m6A.IMPACT.genes	24h	2113
LNCaP	m6A.IMPACT.genes	48h	188

Supplementary Table 2B. Frequency of miR-96 and m6A-miR-96 targeted genes by time and cell background. The frequency of genes containing miR-96 binding sites (IMPACT.genes) and those shared with m6A (m6A.IMPACT.genes) were calculated at the indicated time points in each cell backgroun (n).

IMPACT-Seq: Coregulators and TFs

	HPr1AR*24h* IMP N = 472[1]	HPr1AR*24h* m6A.IMP N = 241[1]	LNCaP*48h* IMP N = 333[1]	LNCaP*48h* m6A.IMP N = 263[1]	**p-value**[2]
**CoA**	32 (6.8%)	14 (5.8%)	15 (4.5%)	10 (3.8%)	0.3
**CoR**	16 (3.4%)	8 (3.3%)	11 (3.3%)	13 (4.9%)	0.7
**Mixed**	17 (3.6%)	9 (3.7%)	7 (2.1%)	4 (1.5%)	0.3
**TF**	41 (8.7%)	22 (9.1%)	16 (4.8%)	5 (1.9%)	<0.001
**NR**	2 (0.4%)	0 (0%)	0 (0%)	0 (0%)	0.5

[1]n (%)

[2]Fisher's Exact Test for Count Data with simulated p-value (based on 2000 replicates)

IMPACT-Seq: Coregulators and TFs

	HPr1AR24*h* IMP.genes, N = 423[1]	HPr1AR*24h*m6A. IMP.genes, N = 227[1]	**LNCaP*48h* IMP.genes**, N = 245[1]	**LNCaP*48h*m6A. IMP.genes**, N = 188[1]	**p-value**[2]
**CoA**	27 (6.4%)	13 (5.7%)	11 (4.5%)	6 (3.2%)	0.4
**CoR**	13 (3.1%)	8 (3.5%)	9 (3.7%)	8 (4.3%)	0.9
**Mixed**	15 (3.5%)	8 (3.5%)	6 (2.4%)	4 (2.1%)	0.8
**TF**	40 (9.5%)	20 (8.8%)	16 (6.5%)	5 (2.7%)	0.014
**NR**	2 (0.5%)	0 (0%)	0 (0%)	0 (0%)	0.7

[1]n (%)

[2]Fisher's Exact Test for Count Data with simulated p-value (based on 2000 replicates)

Supplementary Table 2C. Enrichment of different classes of protein coding genes in miR-96 targetomes. miR-96 targeted protein coding genes were categorized if they were coactivators (CoAs), corepressors (CoRs), mixed function coregulators (Mixed) or transcription and mRNA stabilization factors (TFs) and the frequency of the number of sites (**Top**) or frequency of targeted genes (**Bottom**) was determined and the significant differences measured with a Fisher's exact test.

Cell	coreg	NR	Direct	HPr1AR. MRE	HPr1AR. m6A.MRE	LNCaP. m6A.MRE	LNCaP. MRE	Most Significant
HPr1AR	CoA	-	N	3	3	0	0	EEF2
HPr1AR	CoA	-	P	7	6	0	0	GRHL3
HPr1AR	CoR	-	N	2	2	0	0	PDCD4
HPr1AR	CoR	-	P	1	1	0	0	SFMBT1
HPr1AR	Mixed	-	N	2	0	0	0	MAGED1
HPr1AR	Mixed	-	N	0	1	0	0	BRPF3
HPr1AR	Mixed	-	P	3	6	0	0	RBPJ
HPr1AR	other	-	N	0	84	0	0	SLC39A1
HPr1AR	other	-	N	112	0	0	0	MSN
HPr1AR	other	-	P	0	64	0	0	CDKN1A
HPr1AR	other	-	P	105	0	0	0	ALPK2
HPr1AR	TF	-	N	6	6	0	0	ZFP36L1
HPr1AR	TF	-	P	14	14	0	0	E2F7
LNCaP	CoA	-	N	0	0	11	14	TACC1
LNCaP	CoA	-	P	0	0	0	10	MYO6
LNCaP	CoA	-	P	0	0	12	0	PPP2R5B
LNCaP	CoR	-	N	0	0	7	0	PDCD4
LNCaP	CoR	-	N	0	0	0	6	CBX6
LNCaP	CoR	-	P	0	0	6	4	TAF9B
LNCaP	Mixed	-	N	0	0	5	7	TBL1X
LNCaP	Mixed	-	P	0	0	4	7	RBPJ
LNCaP	other	-	N	0	0	163	248	CD164
LNCaP	other	-	P	0	0	104	154	CBLN2
LNCaP	TF	-	N	0	0	13	20	CREB3L2
LNCaP	TF	Yes	N	0	0	2	3	RARG
LNCaP	TF	-	P	0	0	11	13	TRIB1

Supplementary Table 3A: Regulated mRNA targets of miR-96 mimics in HPr1AR and LNCaP cells. Cells were transfected with a miR-96 mimic or scrambled control (50nM, 48 h) and RNA-Seq undertaken to identify significantly differentially expressed genes (DEGs), which were then divided in negatively (N) and positively (P) regulated genes and annotated as to whether they were either a coactivator (CoA), corepressor (CoR), mixed function coregulator (mixed) or a transcription factor (TF), and if a TF, whether a nuclear receptor (NR). Genes in each of these groups were then annotated as to whether they were bound by miR-96 (MRE), or at a MRE of significant m6A enrichment (m6A-MRE). Finally, for each grouping the gene with the greatest significance was identified.

Cell	coreg	NR	Direct	HPr1AR. MRE.24	HPr1AR. MRE.48	LNCaP. MRE.24	LNCaP. MRE.48	Most Significant
HPr1AR	CoA	-	N	10	16	0	0	EEF1A1
HPr1AR	CoA	-	P	14	24	0	0	ATAD2
HPr1AR	CoR	-	N	5	9	0	0	HSPA8
HPr1AR	CoR	-	P	0	22	0	0	NELFCD
HPr1AR	CoR	-	P	8	0	0	0	UBE2I
HPr1AR	Mixed	-	N	0	9	0	0	HNRNPC
HPr1AR	Mixed	-	N	7	0	0	0	PHF6
HPr1AR	Mixed	-	P	4	16	0	0	NIPBL
HPr1AR	other	-	N	113	0	0	0	PKM
HPr1AR	other	-	N	0	169	0	0	CFL1
HPr1AR	other	-	P	0	255	0	0	VPS37B
HPr1AR	other	-	P	172	0	0	0	UBE2D3
HPr1AR	TF	-	N	8	11	0	0	POLR2A
HPr1AR	TF	-	P	9	15	0	0	SUPT5H
HPr1AR	TF	NR	P	1	1	0	0	NR3C1
LNCaP	CoA	-	N	0	0	9	0	EEF1A1
LNCaP	CoA	-	N	0	0	0	1	MORF4L2
LNCaP	CoA	-	P	0	0	8	0	HAX1
LNCaP	CoA	-	P	0	0	0	2	BAZ2A
LNCaP	CoR	-	N	0	0	2	0	HSPA8
LNCaP	CoR	-	P	0	0	3	0	MBD3
LNCaP	Mixed	-	N	0	0	1	0	MECP2
LNCaP	Mixed	-	P	0	0	3	0	GLS
LNCaP	Mixed	-	P	0	0	0	1	CHD8
LNCaP	other	-	N	0	0	82	0	TUBA1B
LNCaP	other	-	N	0	0	0	13	CFL1
LNCaP	other	-	P	0	0	89	0	PTCD3
LNCaP	other	-	P	0	0	0	6	RPL8
LNCaP	TF	-	N	0	0	4	0	NUCKS1
LNCaP	TF	-	N	0	0	0	1	ENO1
LNCaP	TF	-	P	0	0	3	0	CUX1

Supplementary Table 3B: Regulated protein targets of miR-96 mimics in HPr1AR and LNCaP cells. were transfected with a miR-96 mimic or scrambled control (50 nM, 48 h) and label free quantitative proteomics undertaken to identify significantly differentially expressed proteins (DEPs), which were then divided in negatively (N) and positively (P) regulated targets and annotated as to whether they were either a coactivator (CoA), corepressor (CoR), mixed function coregulator (mixed) or a transcription factor (TF), and if a TF, whether a nuclear receptor (NR). Genes in each of these groups were then annotated as to whether they were bound by miR-96 (MRE), or at a MRE of significant m6A enrichment (m6A-MRE). Finally, for each grouping the target with the greatest significance was identified.

Cell.Rx	Direct	IMPACT	Overlap	logPV.hyper	MostSignificant
22RV1.RARg.TACC1_veh	P	HPR.m6A.IMP	250	50.07	PRRC2C
22RV1.RARg_CD437	P	HPR.m6A.IMP	212	48.68	PRRC2C
22RV1.RARg.TACC1_CD437	P	HPR.m6A.IMP	258	48.52	DNMT1
22RV1.RARg_CD437	P	HPR.IMP	208	41.51	PRRC2C
22RV1.RARg.TACC1_CD437	P	HPR.IMP	255	41.48	DNMT1
22RV1.RARg.TACC1_veh	P	HPR.IMP	244	41.39	PRRC2C
22RV1.RARg_veh	P	HPR.m6A.IMP	141	37.22	SSRP1
22RV1.RARg_veh	P	HPR.IMP	141	33.97	SSRP1
LNCaP_CD437	P	HPR.IMP	98	26.70	NSUN2
LNCaP_veh	P	HPR.m6A.IMP	115	24.86	NSUN2
LNCaP_veh	P	HPR.IMP	114	21.81	NSUN2
LNCaP_CD437	P	HPR.m6A.IMP	87	21.18	NSUN2
22RV1.RARg_CD437	P	LNC.IMP	136	16.58	RARG
22RV1.RARg.TACC1_veh	P	LNC.m6A.IMP	143	14.93	RARG
22RV1.RARg.TACC1_CD437	P	LNC.m6A.IMP	146	13.59	RARG
22RV1.RARg_CD437	P	LNC.m6A.IMP	117	13.53	RARG
LNCaP_CD437	P	LNC.IMP	67	13.11	RARG
22RV1.RARg.TACC1_CD437	P	LNC.IMP	159	13.01	RARG
22RV1.RARg_veh	P	LNC.m6A.IMP	81	12.44	RARG
22RV1.RARg.TACC1_veh	P	LNC.IMP	145	10.90	RARG
22RV1.RARg_veh	P	LNC.IMP	84	10.62	RARG
LNCaP_veh	P	LNC.m6A.IMP	72	10.50	RARG
LNCaP_CD437	P	LNC.m6A.IMP	50	7.15	RARG
LNCaP_veh	P	LNC.IMP	69	6.78	RARG

Supplementary Table 3C: Enrichment of miR-96 MRE targets in the RARγ complex**.** LNCaP, 22Rv1, 22Rv1-RARγ and 22Rv1-RARγ-TACC1 cells in quadruplicate were treated with vehicle or CD437 (400 nM, 6 h) and RIME undertaken to identify significantly and positively enriched proteins in the complex, which were then annotated as to whether they enriched for proteins encoded by MRE-containing genes, and the RARγ complexes are ranked, and the most positively enriched protein is indicated.

Cell.Rx	Direct	coreg	Number	MostSignificant
LNCaP.CD437 vs veh	N	Coactivator	9	PTBP1
LNCaP.CD437 vs veh	N	Corepressor	6	NCOR2
LNCaP.CD437 vs veh	N	Mixed	11	HNRNPA1
LNCaP.CD437 vs veh	P	Coactivator	49	KMT2D
LNCaP.CD437 vs veh	P	Corepressor	21	RCOR3
LNCaP.CD437 vs veh	P	Mixed	26	SRC
LNCaP.CD437 vs veh	P	TF	51	RXRA
RV1.RARg.CD437 vs veh	P	Corepressor	1	CBX4
RV1.RARg.CD437 vs veh	P	TF	2	SUPT5H
RV1 vs LNCaP RARg.veh	N	Coactivator	6	MYO6
RV1 vs LNCaP RARg.veh	N	Corepressor	3	NCOR2
RV1 vs LNCaP RARg.veh	N	Mixed	1	HNRNPA0
RV1 vs LNCaP RARg.veh	N	TF	2	POLR1C
RV1 vs LNCaP RARg.veh	P	Coactivator	37	PHIP
RV1 vs LNCaP RARg.veh	P	Corepressor	16	KHDRBS1
RV1 vs LNCaP RARg.veh	P	Mixed	23	HNRNPH3
RV1 vs LNCaP RARg.veh	P	TF	31	ZBTB10
RV1.RARg.TACC1.CD437 vs veh	N	Coactivator	2	NSD1
RV1.RARg.TACC1.CD437 vs veh	N	Corepressor	2	NCOR2

Supplementary Table 3D: Change in enrichment of coregulators in the RARγ complex**.** LNCaP, 22Rv1, 22Rv1-RARγ and 22Rv1-RARγ-TACC1 cells in quadruplicate were treated with vehicle or CD437 (400 nM, 6 h) and RIME undertaken. In each case the change in enrichment was then calculated between the indicated conditions to identify differentially enriched proteins, both negatively and positively enriched in the complex, which were then annotated as to whether they were either a coactivator (CoA), corepressor (CoR), mixed function coregulator (mixed) or a transcription factor (TF). A hypergeometric test was undertaken to examine if the overlaps for each group of proteins were significantly enriched in these altered enrichments in the RARγ complex, and the most significantly altered protein is indicated.

Cell.Rx	Coreg	IMPACT	Number	MostSignificant
22RV1.RARg.TACC1_CD437	CoA	HPR.IMP	46	TRRAP
22RV1.RARg.TACC1_veh	CoA	HPR.IMP	46	NSD1
22RV1.RARg.TACC1_veh	CoA	HPR.m6A.IMP	46	NSD1
22RV1.RARg.TACC1_CD437	CoA	HPR.m6A.IMP	45	TRRAP
22RV1.RARg_CD437	CoA	HPR.IMP	36	NSUN2
22RV1.RARg_CD437	CoA	HPR.m6A.IMP	36	NSUN2
22RV1.RARg.TACC1_veh	TF	HPR.m6A.IMP	35	CSDE1
22RV1.RARg.TACC1_CD437	TF	HPR.m6A.IMP	32	SUPT5H
22RV1.RARg.TACC1_veh	TF	HPR.IMP	30	CSDE1
22RV1.RARg.TACC1_CD437	TF	HPR.IMP	28	SUPT5H
22RV1.RARg.TACC1_veh	CoA	LNC.m6A.IMP	28	NSD1
22RV1.RARg_veh	CoA	HPR.m6A.IMP	26	NSUN2
22RV1.RARg.TACC1_CD437	CoA	LNC.m6A.IMP	26	TRRAP
22RV1.RARg.TACC1_CD437	CoA	LNC.IMP	25	TRRAP
22RV1.RARg_veh	CoA	HPR.IMP	24	NSUN2
22RV1.RARg.TACC1_veh	CoA	LNC.IMP	24	NSD1
22RV1.RARg.TACC1_CD437	Mixed	HPR.m6A.IMP	23	HCFC1
22RV1.RARg_CD437	TF	HPR.m6A.IMP	22	DDB1
22RV1.RARg_CD437	CoA	LNC.IMP	21	SUPT16H
22RV1.RARg.TACC1_CD437	Mixed	HPR.IMP	21	HCFC1
22RV1.RARg.TACC1_veh	CoR	HPR.m6A.IMP	21	DNMT1
22RV1.RARg.TACC1_veh	Mixed	HPR.m6A.IMP	21	HCFC1
22RV1.RARg_CD437	CoA	LNC.m6A.IMP	20	SUPT16H
22RV1.RARg_CD437	TF	HPR.IMP	20	DDB1
22RV1.RARg.TACC1_veh	Mixed	HPR.IMP	20	HCFC1
22RV1.RARg.TACC1_CD437	CoR	HPR.m6A.IMP	19	DNMT1
22RV1.RARg.TACC1_veh	CoR	HPR.IMP	19	DNMT1
LNCaP_veh	CoA	HPR.m6A.IMP	19	NSUN2
LNCaP_veh	CoA	HPR.IMP	18	NSUN2
22RV1.RARg.TACC1_CD437	CoR	HPR.IMP	17	DNMT1
22RV1.RARg_CD437	CoR	HPR.m6A.IMP	16	XRCC5
22RV1.RARg_veh	CoA	LNC.m6A.IMP	16	SUPT16H
22RV1.RARg.TACC1_veh	TF	LNC.IMP	16	RARG
22RV1.RARg_CD437	Mixed	HPR.m6A.IMP	15	HCFC1
22RV1.RARg.TACC1_CD437	CoR	LNC.IMP	15	XRCC5
22RV1.RARg.TACC1_CD437	Mixed	LNC.IMP	15	HCFC1
22RV1.RARg.TACC1_CD437	TF	LNC.IMP	15	RARG
22RV1.RARg.TACC1_veh	TF	LNC.m6A.IMP	15	RARG
22RV1.RARg_CD437	CoR	HPR.IMP	14	XRCC5
22RV1.RARg_veh	CoA	LNC.IMP	14	SUPT16H
22RV1.RARg.TACC1_CD437	CoR	LNC.m6A.IMP	14	XRCC5
22RV1.RARg.TACC1_veh	CoR	LNC.m6A.IMP	14	NACC1
LNCaP_CD437	CoA	HPR.m6A.IMP	14	NSUN2
22RV1.RARg_CD437	Mixed	HPR.IMP	13	HCFC1
22RV1.RARg.TACC1_CD437	Mixed	LNC.m6A.IMP	13	HCFC1
22RV1.RARg.TACC1_CD437	TF	LNC.m6A.IMP	13	RARG
22RV1.RARg.TACC1_veh	CoR	LNC.IMP	13	NACC1
22RV1.RARg.TACC1_veh	Mixed	LNC.IMP	13	HCFC1
LNCaP_CD437	CoA	HPR.IMP	13	NSUN2
22RV1.RARg_CD437	TF	LNC.IMP	12	RARG
22RV1.RARg_veh	CoR	HPR.m6A.IMP	12	DNMT1

22RV1.RARg.TACC1_veh	Mixed	LNC.m6A.IMP	12	HCFC1
LNCaP_veh	CoA	LNC.IMP	12	SUPT16H
22RV1.RARg_CD437	CoR	LNC.IMP	11	XRCC5
22RV1.RARg_CD437	CoR	LNC.m6A.IMP	11	XRCC5
22RV1.RARg_CD437	Mixed	LNC.IMP	11	HCFC1
22RV1.RARg_veh	CoR	HPR.IMP	11	DNMT1
22RV1.RARg_veh	TF	HPR.m6A.IMP	11	DDB1
LNCaP_veh	CoA	LNC.m6A.IMP	11	SUPT16H

Supplementary Table 3D– Change in enrichment of coregulators that contain a miR-96 MRE in the RARγ complex. LNCaP, 22Rv1, 22Rv1-RARγ and 22Rv1-RARγ-TACC1 cells in quadruplicate were treated with vehicle or CD437 (400 nM, 6 h) and RIME undertaken. In each case enriched proteins, compared to IgG, were annotated as to whether they were either a coactivator (CoA), corepressor (CoR), mixed function coregulator (mixed) or a transcription factor (TF) and contained a miR-96 binding site by IMPACT-Seq (IMP) that also contained a m6A modified region (m6A.IMP) in the indicated conditions. The number of proteins that meet each criterion is indicated and the most positively enriched protein is indicated.

Cell	Target	Rx	n	logPval
Mock	AR	veh	768	260.81
Mock	AR	DHT	1368	64.36
RARg	AR	veh	9649	291.65
RARg	AR	DHT	5554	330.78
TACC1.RARg	AR	veh	82090	375.79
TACC1.RARg	AR	DHT	119149	387.00
RARg	RARγ	veh	411	137.44
RARg	RARγ	CD437	284	402.08
TACC1.RARg	RARγ	veh	6278	455.64
TACC1.RARg	RARγ	CD437	378	332.87
Mock	K27	veh	29755	628.63
Mock	K27	DHT	44594	612.61
RARg	K27	veh	26554	647.89
RARg	K27	DHT	25446	658.48
RARg	K27	CD437	25838	652.64
TACC1.RARg	K27	veh	43758	675.59
TACC1.RARg	K27	DHT	29118	657.20
TACC1.RARg	K27	CD437	32828	621.60

Supplementary Table 4A. Summary of significantly enriched binding sites for AR, RARγ and H3K27ac. 22Rv1, 22Rv1-RARγ and 22Rv1-RARγ-TACC1 cells in triplicate were treated for 6 h with DHT (10nM), CD437 (400 nM) or vehicle, and CUT&RUN undertaken for AR, RARγ or H3K27ac. The number of differentially enriched peaks compared to IgG (n) was determined with `csaw`, and the mean logPV (calculated as -log10(p.adj) for each group) are indicated.

Cell.ChIP.Rx	ChromHMM	logPV	Threshold
Mock.K27.DHT	Promoter	317.17	Significant
Mock.K27.DHT	Transcribed	317.17	Significant
Mock.K27.veh	Promoter	317.17	Significant
Mock.K27.veh	Transcribed	317.17	Significant
RARg.AR.DHT	Promoter	317.17	Significant
RARg.AR.veh	Promoter	317.17	Significant
RARg.K27.veh	Promoter	317.17	Significant
RARg.K27.DHT	Promoter	317.17	Significant
RARg.K27.CD437	Promoter	317.17	Significant
RARg.K27.CD437	Transcribed	317.17	Significant
TACC1.RARg.AR.DHT	Transcribed	317.17	Significant
TACC1.RARg.AR.DHT	Poised_Enhancer	317.17	Significant
TACC1.RARg.AR.DHT	Promoter	317.17	Significant
TACC1.RARg.AR.DHT	Active_Enhancer	317.17	Significant
TACC1.RARg.AR.DHT	Polycomb	317.17	Significant
TACC1.RARg.K27.CD437	Promoter	317.17	Significant
TACC1.RARg.K27.CD437	Transcribed	317.17	Significant
TACC1.RARg.K27.veh	Promoter	317.17	Significant
TACC1.RARg.K27.veh	Active_Enhancer	317.17	Significant
TACC1.RARg.K27.veh	Transcribed	317.17	Significant
TACC1.RARg.K27.DHT	Promoter	317.17	Significant
TACC1.RARg.K27.DHT	Transcribed	317.17	Significant
TACC1.RARg.K27.DHT	Active_Enhancer	317.17	Significant
TACC1.RARg.RARg.veh	Promoter	317.17	Significant
TACC1.RARg.RARg.veh	Poised_Enhancer	317.17	Significant
TACC1.RARg.RARg.veh	Active_Enhancer	317.17	Significant
TACC1.RARg.RARg.veh	Transcribed	317.17	Significant
TACC1.RARg.K27.CD437	Active_Enhancer	316.88	Significant
RARg.RARg.veh	Promoter	297.22	Significant
TACC1.RARg.RARg.CD437	Promoter	244.78	Significant
RARg.K27.veh	Transcribed	225.56	Significant
RARg.RARg.CD437	Promoter	207.81	Significant
TACC1.RARg.RARg.veh	Polycomb	143.32	Significant
RARg.K27.DHT	Active_Enhancer	116.49	Significant
RARg.K27.DHT	Transcribed	99.32	Significant
RARg.AR.DHT	Active_Enhancer	98.98	Significant
RARg.K27.CD437	Active_Enhancer	98.22	Significant
TACC1.RARg.AR.DHT	Bivalent_Promoter	93.86	Significant
Mock.AR.veh	Promoter	91.28	Significant
Mock.AR.DHT	Promoter	86.14	Significant
RARg.K27.veh	Active_Enhancer	80.35	Significant
Mock.K27.veh	Active_Enhancer	60.28	Significant
RARg.AR.veh	Active_Enhancer	55.61	Significant
Mock.AR.DHT	Active_Enhancer	49.43	Significant
Mock.K27.DHT	Active_Enhancer	46.12	Significant
TACC1.RARg.RARg.veh	Bivalent_Promoter	5.63	Significant
Mock.AR.veh	Active_Enhancer	2.37	Significant

Supplementary Table 4B: Significant enrichment of AR, RARγ and H3K27ac cistromes in ChromHMM defined epigenetic states. 22Rv1-mock, 22Rv1-RARγ and 22Rv1-RARγ-TACC1 cells in triplicate were treated for 6h with vehicle DHT (10nM), or CD437 (400 nM), and CUT&RUN undertaken for AR, RARγ or H3K27ac. Differential enriched binding sites compared to IgG controls (p.adj < .1) were identified by `csaw` and were overlapped using `bedtools` with ChromHMM regions (ChromHMM) identified in LNCaP, and enrichment tested with a hypergeometric test (lower.tail = FALSE).

Cell.Rx	class	coreg.freq	Nearest.Example
Mock_DHT	other	470	LPAR3
Mock_DHT	CoR	13	SNX6
Mock_DHT	TF	12	CASZ1
Mock_DHT	Mixed	10	ZBTB16
Mock_DHT	CoA	6	ZBTB7B
Mock_RARg_DHT	other	45	LPAR3
Mock_RARg_DHT	TF	2	NR4A1
Mock_RARg_DHT	CoR	1	SNX6
Mock_RARg_M	other	30	MYBPC1
Mock_RARg_M	TF	2	NR4A1
Mock_RARg_TACC1.RARg_DHT	other	52	RP11-122G18.12
Mock_RARg_TACC1.RARg_DHT	TF	1	THRB
Mock_RARg_TACC1.RARg_M	other	12	REXO1L7P
Mock_TACC1.RARg_DHT	other	22	KLK15
Mock_TACC1.RARg_DHT	Mixed	1	ZBTB16
Mock_TACC1.RARg_M	other	71	RP11-122G18.12
Mock_TACC1.RARg_M	TF	3	THRB
Mock_TACC1.RARg_M	Mixed	1	ZBTB16
RARg_DHT	other	48	RP11-554D13.1
RARg_DHT	Mixed	3	HFE2
RARg_DHT	CoR	2	CCND1
RARg_DHT	CoA	1	BMPR1B
RARg_DHT	TF	1	FOXA1
RARg_M	other	46	RP11-554D13.1
RARg_M	CoR	2	TXNIP
RARg_M	Mixed	2	HFE2
RARg_M	TF	2	HIST2H2BE
RARg_M	CoA	2	SF3B4
TACC1.RARg_DHT	other	53	DBET
TACC1.RARg_DHT	TF	20	DUX4L8
TACC1.RARg_M	other	123	REXO1L9P
TACC1.RARg_M	TF	8	FOXA1
TACC1.RARg_M	CoR	5	CCND1
TACC1.RARg_M	Mixed	3	NFIX
TACC1.RARg_M	CoA	1	KDM6B
TACC1.RARg_RARg_DHT	other	21	REXO1L8P
TACC1.RARg_RARg_DHT	TF	1	PPARA
TACC1.RARg_RARg_M	other	36	REXO1L8P
TACC1.RARg_RARg_M	TF	5	ZNF565

Supplementary Table 4C: Coregulator and transcription factor annotation of genes associated with super enhancers. 22Rv1, 22Rv1-RARγ and 22Rv1-RARγ-TACC1 cells in triplicate were treated with vehicle or DHT (10nM, 6h) or vehicle, and CUT&RUN undertaken for H3K27ac, and differentially-enriched sites were analyzed, including those at the sites of shared binding, with the `ROSE` algorithm and using BRD4 as a enhancer-enriched factor to generate high confidence super enhancer sites (SE). The SE were annotated to genes within 100 kb and classified to identify coregulators (CoA, CoR, Mixed) and TFs, or other. The closest example is shown in each cell, treatment and coregulator class.

Bkgd	ChIP	Rx	n	logPval
Syn	AR	DHT	47695	1.24
Syn	AR	veh	30	1.66
Asy	AR	DHT	15545	1.54
Asy	AR	veh	6354	1.26
Syn	RARγ	DHT	2035	1.27
Syn	RARγ	veh	2037	1.42
Syn	H3S10	DHT	96123	3.60
Syn	H3S10	veh	151480	2.25
Asy	H3S10	veh	1634	2.13
Asy	RARγ	DHT	388	1.41
Asy	RARγ	veh	295	1.41

Supplementary Table 5: Cistrome in G2/M synchronized and asynchronized cells. 22Rv1-RARγ cells in triplicate were treated with vehicle or nocodozole (*60 ng*, 18h) and then (DHT (10nM, 6 h) or vehicle, and CUT&RUN undertaken for AR, RARγ and H3S10P, and differentially-enriched sites identified with `csaw`. The number of differentially enriched peaks compared to IgG (n), and the mean logPV (calculated as -log10(p.adj) for each group are indicated.

Gen.Rx	D	MostSig	Lum	Bas	CoA	CoR	Mix	TF	NR	in.miR	MRE
Mock_CD437	N	YAP1	0	0	0	0	62	0	0	0	0
Mock_CD437	N	PEG10	0	128	0	0	0	0	0	0	0
Mock_CD437	P	DHRS3	0	0	0	0	0	0	0	126	107
Mock_CD437	P	EPAS1	0	0	0	0	0	159	0	0	0
Mock_CD437	P	NR6A1	0	0	0	0	0	159	9	0	0
Mock_CD437	P	BTG2	0	0	0	47	0	0	0	0	0
Mock_CD437	P	BAMBI	188	0	73	0	0	0	0	0	0
Mock_DHT	P	SHROOM3	260	0	0	0	0	0	0	153	124
Mock_DHT	P	CEBPD	0	0	0	0	0	206	0	0	0
Mock_DHT	P	NT5DC3	0	155	0	0	0	0	0	0	0
Mock_DHT	P	YAP1	0	0	0	0	61	0	0	0	0
Mock_DHT	P	CD36	0	0	0	60	0	0	0	0	0
Mock_DHT	P	FZD5	0	0	88	0	0	0	0	0	0
Mock_DHT	P	RARB	0	0	0	0	0	0	10	0	0
Mock_ENZA	N	BTG1	0	0	0	0	0	141	0	0	0
Mock_ENZA	N	TMEFF2	163	0	0	0	0	0	0	0	0
Mock_ENZA	N	STEAP2	0	0	0	0	0	0	0	0	89
Mock_ENZA	N	BARD1	0	0	0	0	50	0	0	0	0
Mock_ENZA	N	NR3C2	0	0	0	0	0	0	13	0	0
Mock_ENZA	P	GALNT10	0	0	0	0	0	0	0	106	0
Mock_ENZA	P	PRKD1	0	0	60	0	0	0	0	0	0
Mock_ENZA	P	TOB1	0	0	0	29	0	0	0	0	0
Mock_ENZA	P	SPRY2	0	92	0	0	0	0	0	0	0
RARg_CD437	N	KLHL29	0	128	0	0	0	0	0	0	0
RARg_CD437	P	SHROOM1	184	0	0	0	0	0	0	0	0
RARg_CD437	P	DHRS3	0	0	0	0	0	0	0	124	112
RARg_CD437	P	EPAS1	0	0	0	0	0	199	0	0	0
RARg_CD437	P	BAMBI	0	0	77	0	0	0	0	0	0
RARg_CD437	P	NRIP1	0	0	0	0	64	0	0	0	0
RARg_CD437	P	BTG2	0	0	0	44	0	0	0	0	0
RARg_CD437	P	NR6A1	0	0	0	0	0	0	20	0	0
RARg_DHT	P	ELOVL2	186	0	0	0	0	0	0	0	0
RARg_DHT	P	NT5DC3	0	97	0	0	0	0	0	0	0
RARg_DHT	P	KLF9	0	0	0	0	0	120	0	0	0
RARg_DHT	P	TMEM164	0	0	0	0	0	0	0	100	0
RARg_DHT	P	SHROOM3	0	0	0	0	0	0	0	0	92
RARg_DHT	P	FZD5	0	0	49	0	0	0	0	0	0
RARg_DHT	P	FZD1	0	0	0	0	33	0	0	0	0
RARg_DHT	P	TFCP2L1	0	0	0	41	0	0	0	0	0
RARg_DHT	P	RARB	0	0	0	0	0	0	3	0	0
RARg_ENZA	N	DKK1	0	62	0	26	0	0	0	0	0
RARg_ENZA	P	DHRS3	0	0	0	0	0	0	0	82	63
RARg_ENZA	P	ATP1B1	123	0	0	0	0	0	0	0	0
RARg_ENZA	P	BAMBI	123	0	34	0	0	0	0	0	0
RARg_ENZA	P	EPAS1	0	0	0	0	0	111	0	0	0
RARg_ENZA	P	NRIP1	0	0	0	0	28	0	0	0	0
RARg_ENZA	P	PPARA	0	0	0	0	0	0	10	0	0
RARg_veh	N	PLEKHG5	0	100	0	0	0	0	0	0	0
RARg_veh	N	AGO1	0	0	44	0	0	0	0	0	0
RARg_veh	N	HNF4A	0	0	0	0	0	0	8	0	0
RARg_veh	P	CLU	0	0	0	0	0	0	0	79	0
RARg_veh	P	SOX9	0	0	0	0	0	119	0	0	71

RARg_veh	P	SLC12A2	139	0	0	0	0	0	0	0	0
RARg_veh	P	LEF1	0	0	0	0	33	0	0	0	0
RARg_veh	P	PPID	0	0	0	25	0	0	0	0	0
TACC1.RARg_CD437	P	SHROOM1	261	0	0	0	0	0	0	0	0
TACC1.RARg_CD437	P	ABLIM3	0	0	136	0	0	0	0	0	0
TACC1.RARg_CD437	P	NRIP1	0	0	0	0	95	0	0	0	0
TACC1.RARg_CD437	P	CORO2A	0	0	0	86	0	0	0	0	0
TACC1.RARg_CD437	P	PPARD	0	0	0	0	0	305	19	0	0
TACC1.RARg_CD437	P	EPAS1	0	0	0	0	0	0	0	0	178
TACC1.RARg_CD437	P	UPP1	0	191	0	0	0	0	0	0	0
TACC1.RARg_CD437	P	DHRS3	0	0	0	0	0	0	0	226	0
TACC1.RARg_DHT	P	ELOVL2	110	0	0	0	0	0	0	0	0
TACC1.RARg_DHT	P	KCNJ11	110	0	0	0	0	0	0	0	0
TACC1.RARg_DHT	P	TFEB	0	0	0	0	0	59	0	0	0
TACC1.RARg_DHT	P	NT5DC3	0	40	0	0	0	0	0	0	0
TACC1.RARg_DHT	P	FSTL1	0	0	0	0	0	0	0	61	0
TACC1.RARg_DHT	P	SHROOM3	0	0	0	0	0	0	0	0	52
TACC1.RARg_DHT	P	YAP1	0	0	0	0	21	0	0	0	0
TACC1.RARg_DHT	P	FZD5	0	0	26	0	0	0	0	0	0
TACC1.RARg_DHT	P	RARB	0	0	0	0	0	0	3	0	0
TACC1.RARg_DHT	P	PIAS1	0	0	0	20	0	0	0	0	0
TACC1.RARg_ENZA	N	DKK1	0	16	0	0	0	0	0	0	0
TACC1.RARg_ENZA	P	CORO2A	0	0	0	11	0	0	0	0	0
TACC1.RARg_ENZA	P	SHROOM1	72	0	0	0	0	0	0	0	0
TACC1.RARg_ENZA	P	ABLIM3	0	0	15	0	0	0	0	0	0
TACC1.RARg_ENZA	P	NRIP1	0	0	0	0	12	0	0	0	0
TACC1.RARg_ENZA	P	DHRS3	0	0	0	0	0	0	0	29	37
TACC1.RARg_ENZA	P	PPARD	0	0	0	0	0	39	6	0	0
TACC1.RARg_veh	N	KNTC1	0	0	0	0	0	352	0	0	0
TACC1.RARg_veh	N	SMC4	0	0	0	0	0	0	0	224	0
TACC1.RARg_veh	N	ZNF367	0	0	0	0	0	0	0	0	217
TACC1.RARg_veh	N	TMEM237	0	194	0	0	0	0	0	0	0
TACC1.RARg_veh	N	CDK2	0	0	143	0	0	0	0	0	0
TACC1.RARg_veh	N	TIMELESS	0	0	0	108	0	0	0	0	0
TACC1.RARg_veh	N	BRIP1	0	0	0	0	99	0	0	0	0
TACC1.RARg_veh	P	SUSD2	295	0	0	0	0	0	0	0	0
TACC1.RARg_veh	P	RARG	0	0	0	0	0	0	16	0	0

Supplementary Table 6A – **Categorization of DEGs.** LNCaP, 22Rv1-Mock, 22Rv1-RARγ and 22Rv1-RARγ-TACC1 cells in triplicate were treated with vehicle or DHT (10nM), ENZA (10mM), or CD437 (400nM) for 24 h and RNA-Seq undertaken. Each DEG list was annotated as to whether the gene was contained within a luminal (Lum) or basal prostate gene signature (Bas), a coactivator (CoA), corepressor (CoR), mixed function coregulator (mixed), a transcription factor (TF), a nuclear receptor (NR), an intron-encoded miRNA (in.miR) or contained a miR-96 binding site identified by IMPACT-Seq and was differentially expressed either at the mRNA or protein level (MRE). The number of genes in each category, in each cell background and treatment is indicated, and the most significant gene in each class given.

Gen.Rx	Overlap	logPV	Threshold
RARg_CD437	20	4.85	Significant
TACC1.RARg_ENZA	6	2.59	Significant
Mock_ENZA	13	2.24	Significant
RARg_ENZA	10	1.80	Significant
TACC1.RARg_CD437	19	1.68	Significant
RARg_veh	8	1.03	Significant
Mock_DHT	10	0.43	NS
Mock_CD437	9	0.39	NS
TACC1.RARg_DHT	3	0.24	NS
TACC1.RARg_veh	16	0.19	NS
RARg_DHT	3	0.04	NS

Supplementary Table 6B – Enrichment of NRs in DEGs. LNCaP, 22Rv1-Mock, 22Rv1-RARγ and 22Rv1-RARγ-TACC1 cells in triplicate were treated with vehicle or DHT (10nM), ENZA (10mM), or CD437 (400nM) for 24 h and RNA-Seq undertaken. Each DEG list was annotated to identify nuclear receptors (NR). A hypergeometric test was undertaken to examine if the number of NR were significantly enriched in each DEGs list.

Gen.Rx	Overlap	logPV	Threshold
TACC1.RARg_ENZA	37	3.94	Significant
RARg_DHT	92	2.11	Significant
TACC1.RARg_DHT	52	1.39	Significant
TACC1.RARg_CD437	178	0.72	NS
RARg_CD437	112	0.71	NS
RARg_veh	71	0.64	NS
Mock_ENZA	89	0.51	NS
Mock_DHT	124	0.44	NS
RARg_ENZA	63	0.22	NS
Mock_CD437	107	0.18	NS
TACC1.RARg_veh	217	0.04	NS

Supplementary Table 6C – Enrichment of miR96 bound and regulated genes in DEGs. LNCaP, 22Rv1, 22Rv1-RARγ and 22Rv1-RARγ-TACC1 cells in triplicate were treated with vehicle or DHT (10nM), ENZA (10mM), or CD437 (400nM) for 24 h and RNA-Seq undertaken. Each DEG list was annotated to identify miR96 bound and regulated genes. A hypergeometric test was undertaken to examine if the number of miR96 bound and regulated genes were significantly enriched in each DEGs list.

Cell.Rx	class	NumberGenes	MostSignificant
Mock_DHT	other	127	CNTROB
Mock_DHT	TF	13	TFEB
Mock_DHT	miR96.target	7	SEMA3C
Mock_DHT	Mixed	5	TANC2
Mock_DHT	intragenic	5	WIZ
Mock_DHT	CoA	4	SMARCE1
Mock_DHT	CoR	2	SIRT7
Mock_DHT	NR	1	NR1H3
Mock_ENZA	other	207	CNTROB
Mock_ENZA	TF	20	AR
Mock_ENZA	CoA	17	SMARCE1
Mock_ENZA	intragenic	13	AATK
Mock_ENZA	Mixed	7	UHRF2
Mock_ENZA	miR96.target	6	PLA2G6
Mock_ENZA	CoR	5	PCGF2
Mock_ENZA	NR	2	AR
Mock_CD437	other	105	PI4KAP2
Mock_CD437	CoA	8	MED12L
Mock_CD437	CoR	6	NCOR2
Mock_CD437	intragenic	6	NCOR2
Mock_CD437	miR96.target	4	JPT2
Mock_CD437	TF	2	RERE
Mock_CD437	Mixed	1	KRIT1
RARg_DHT	other	109	DLD
RARg_DHT	TF	13	SOX13
RARg_DHT	CoR	6	GFI1
RARg_DHT	intragenic	6	MAD1L1
RARg_DHT	miR96.target	4	SEMA3C
RARg_DHT	Mixed	4	ASXL1
RARg_DHT	CoA	2	KAT5
RARg_ENZA	other	131	SLC25A36
RARg_ENZA	TF	11	AR
RARg_ENZA	intragenic	9	MAD1L1
RARg_ENZA	CoA	6	HYAL2
RARg_ENZA	miR96.target	6	ARL1
RARg_ENZA	CoR	5	NCOR2
RARg_ENZA	Mixed	3	TBL1XR1
RARg_ENZA	NR	1	AR
RARg_CD437	other	477	TBL1XR1
RARg_CD437	CoA	40	SMARCA4
RARg_CD437	TF	37	ZNF544
RARg_CD437	intragenic	36	TLE3
RARg_CD437	miR96.target	19	ARPC1B
RARg_CD437	CoR	16	TLE3
RARg_CD437	Mixed	12	TBL1XR1
RARg_CD437	NR	3	NR1H3
RARg.Mock_DHT	other	157	SPAG5
RARg.Mock_DHT	TF	16	AR
RARg.Mock_DHT	intragenic	10	UHRF1
RARg.Mock_DHT	Mixed	8	CHD8
RARg.Mock_DHT	miR96.target	7	UHRF1
RARg.Mock_DHT	CoR	5	UHRF1

RARg.Mock_DHT	CoA	3	SMARCD3
RARg.Mock_DHT	NR	2	AR
RARg.Mock_ENZA	other	153	SPAG5
RARg.Mock_ENZA	TF	14	RARG
RARg.Mock_ENZA	intragenic	9	ABR
RARg.Mock_ENZA	miR96.target	8	RARG
RARg.Mock_ENZA	CoA	6	PRMT2
RARg.Mock_ENZA	Mixed	4	UHRF2
RARg.Mock_ENZA	NR	2	RARG
RARg.Mock_ENZA	CoR	2	SNX6
RARg.Mock_CD437	other	199	SPAG5
RARg.Mock_CD437	TF	23	AR
RARg.Mock_CD437	intragenic	15	ZBTB20
RARg.Mock_CD437	CoA	10	RRN3
RARg.Mock_CD437	CoR	9	ZBTB20
RARg.Mock_CD437	Mixed	7	TBL1XR1
RARg.Mock_CD437	miR96.target	5	RARG
RARg.Mock_CD437	NR	3	AR

Supplementary Table 6D – Categorization of DETs in 22Rv1 variants. 22Rv1, 22Rv1-RARγ and 22Rv1-RARγ-TACC1 cells in triplicate were treated with DHT (10nM), ENZA (10mM), or CD437 (400nM) for 24 h and RNA-Seq undertaken. FASTQ files were processed with a transcript-aware alignment tool (`Salmon`) and differentially-enriched transcripts (DETs) were identified. The DET lists was annotated to identify coregulators (CoA, CoR, Mixed) and TFs; miR96 bound and regulated genes (miR96.target); or protein-coding genes that contained miRNA (intragenic). The most significant DET in each cell, treatment and category is indicated.

Cell.Rx	class	NumberGenes	MostSignificant
ENZA_miR96	other	82	PROM2
ENZA_miR96	intragenic	9	EVL
ENZA_miR96	CoA	7	NCK1
ENZA_miR96	TF	6	TGIF1
ENZA_miR96	Mixed	5	HNRNPA3
ENZA_miR96	miR96.target	2	DUT
ENZA_miR96	CoR	1	BCLAF1
ENZA_veh	other	10	ENST00000570727
ENZA_veh	TF	2	ZNF136
ENZA_veh	CoA	1	U2AF1
veh_miR96	other	65	EIF3M
veh_miR96	CoA	7	PPP1R12A
veh_miR96	TF	7	TGIF1
veh_miR96	intragenic	5	CPSF6
veh_miR96	miR96.target	3	OAS3
veh_miR96	CoR	2	ELP2
veh_miR96	Mixed	1	ING4

Supplementary Table 7A – Categorization of DETs following miR-96 antagomir plus Enza. 22Rv1 cells in triplicate were treated miR-96 antagomir (50nM) or ENZA (10μM), or the combination or vehicle for 24h and RNA-Seq undertaken. FASTQ files were processed with a transcript-aware alignment tool (`Salmon`) and differentially-enriched transcripts (DETs) were identified. The DET lists was annotated to identify coregulators (CoA, CoR, Mixed) and TFs; miR96 bound and regulated genes (miR96.target); or protein-coding genes that contained miRNA (intragenic). The most significant DET in each cell, treatment and category is indicated.

Cell.Rx	class	hgnc_symbol	logPV	Threshold
ENZA_miR96	CoA	COPA	1.33	Significant
ENZA_miR96	CoA	NCK1	1.33	Significant
ENZA_miR96	CoA	CDC40	1.33	Significant
ENZA_miR96	CoA	TCEA1	1.33	Significant
ENZA_miR96	CoA	BLOC1S2	1.33	Significant
ENZA_miR96	CoA	SMARCE1	1.33	Significant
ENZA_miR96	CoA	SMARCD2	1.33	Significant
ENZA_miR96	CoA	SMARCA4	1.33	Significant

Supplementary Table 7B – Enrichment of functional categories in DETs. 22Rv1 cells in triplicate were treated with vehicle or miR-96 antagomir (50nM) or ENZA (10μM), or the combination for 24h or vehicle, and RNA-Seq undertaken. FASTQ files were processed with a transcript-aware alignment tool (Salmon) and differentially-enriched transcripts (DETs) were identified. The DET lists was annotated to identify coregulators (CoA, CoR, Mixed) and TFs; miR96 bound and regulated genes (miR96.target); or protein-coding genes that contained miRNA (intragenic). A hypergeometric test was undertaken to examine if the number of DETs in each category was significantly enriched, and the results for CoAs, which was significant, are shown.
