# Supplementary material for: Epigenetic disruption of the RARγ complex impairs its function to bookmark AR enhancer interactions required for enzalutamide sensitivity in prostate cancer
Source: bioRxiv. 2023 Dec 19:2023.12.15.571947. Preprint. [Version 2] doi: 10.1101/2023.12.15.571947 (PMC10760102; doi:10.1101/2023.12.15.571947)
Supplement: Supplementary file 1 [file media-1.pdf]

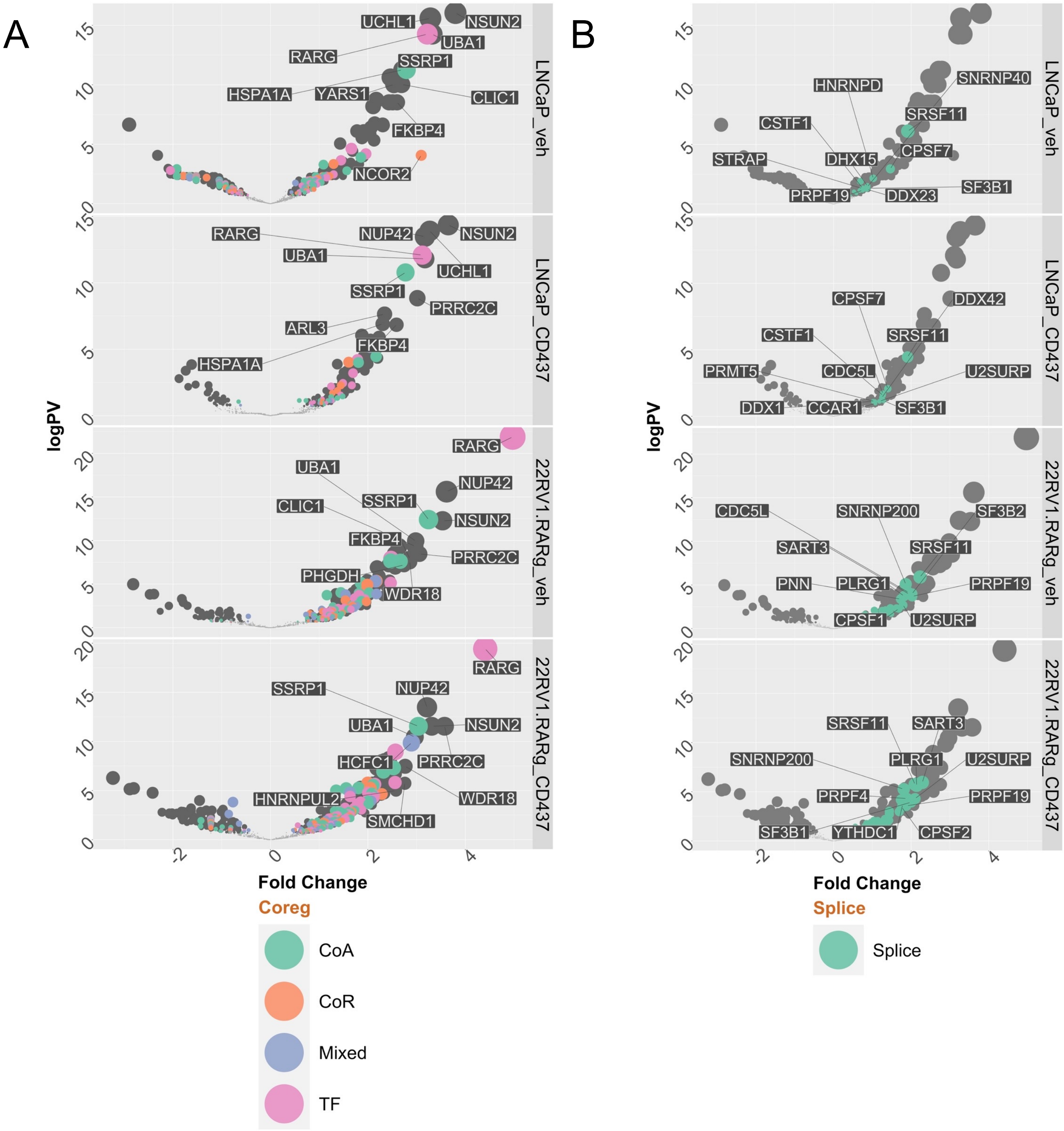

Supplementary Figure 1

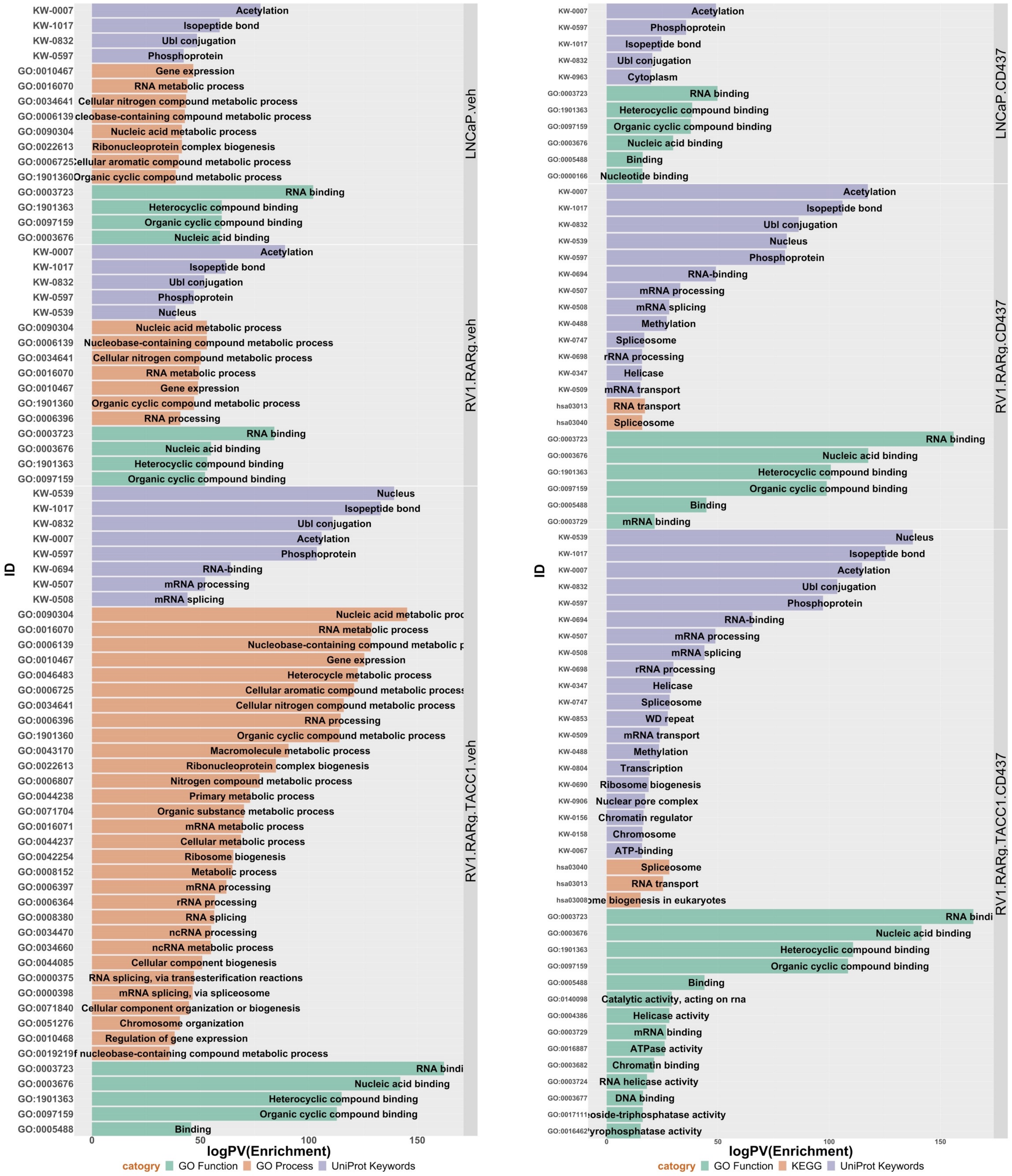

Supplementary Figure 2

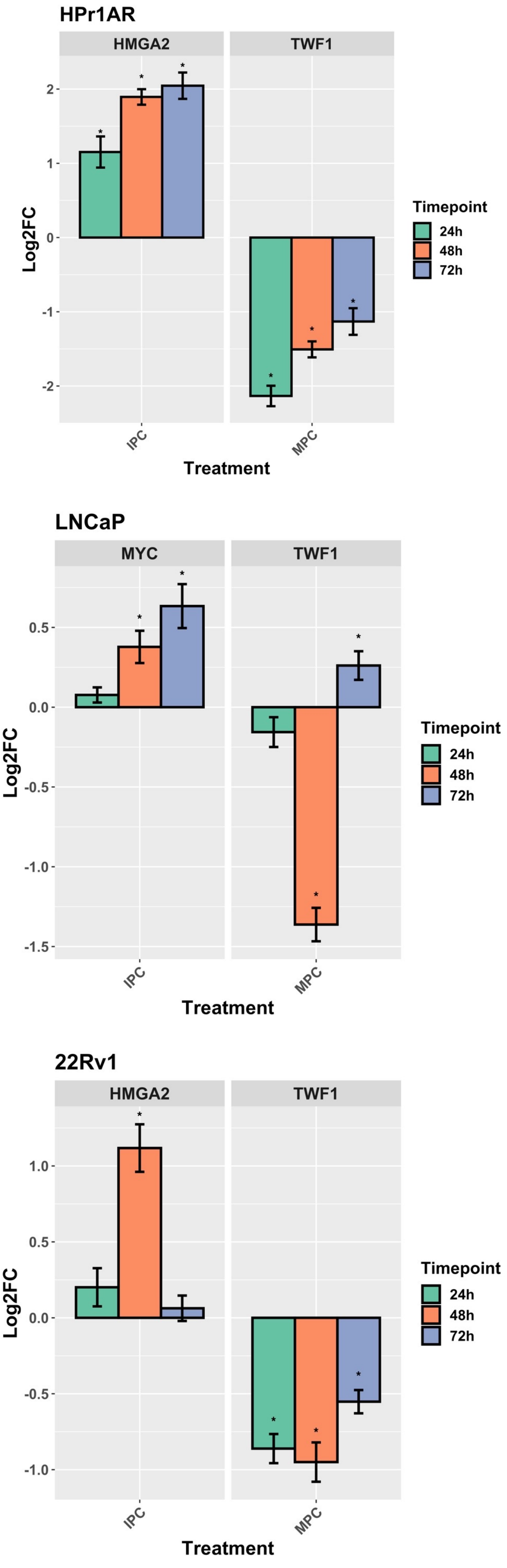

Supplementary Figure 3

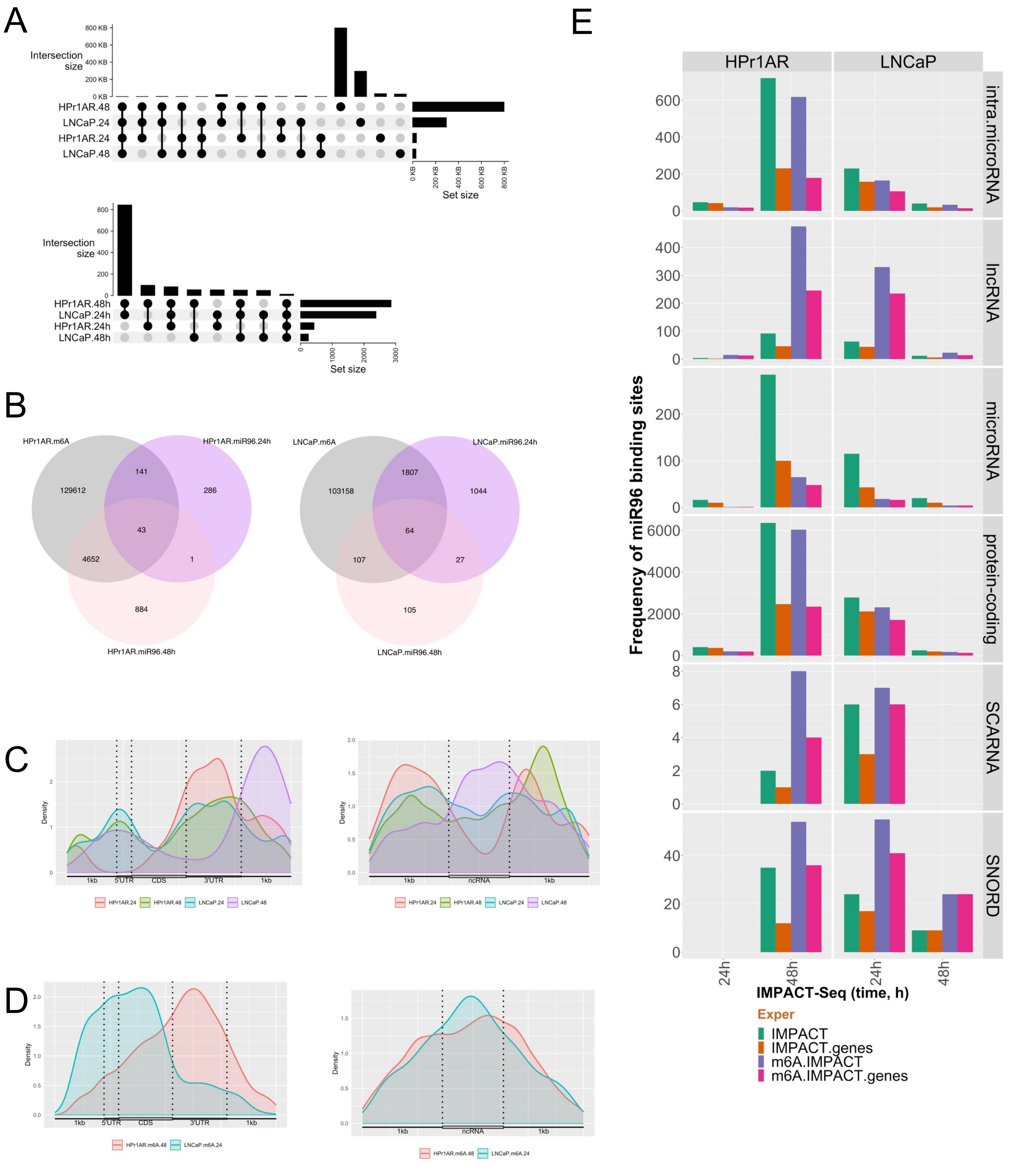

Supplementary Figure 4

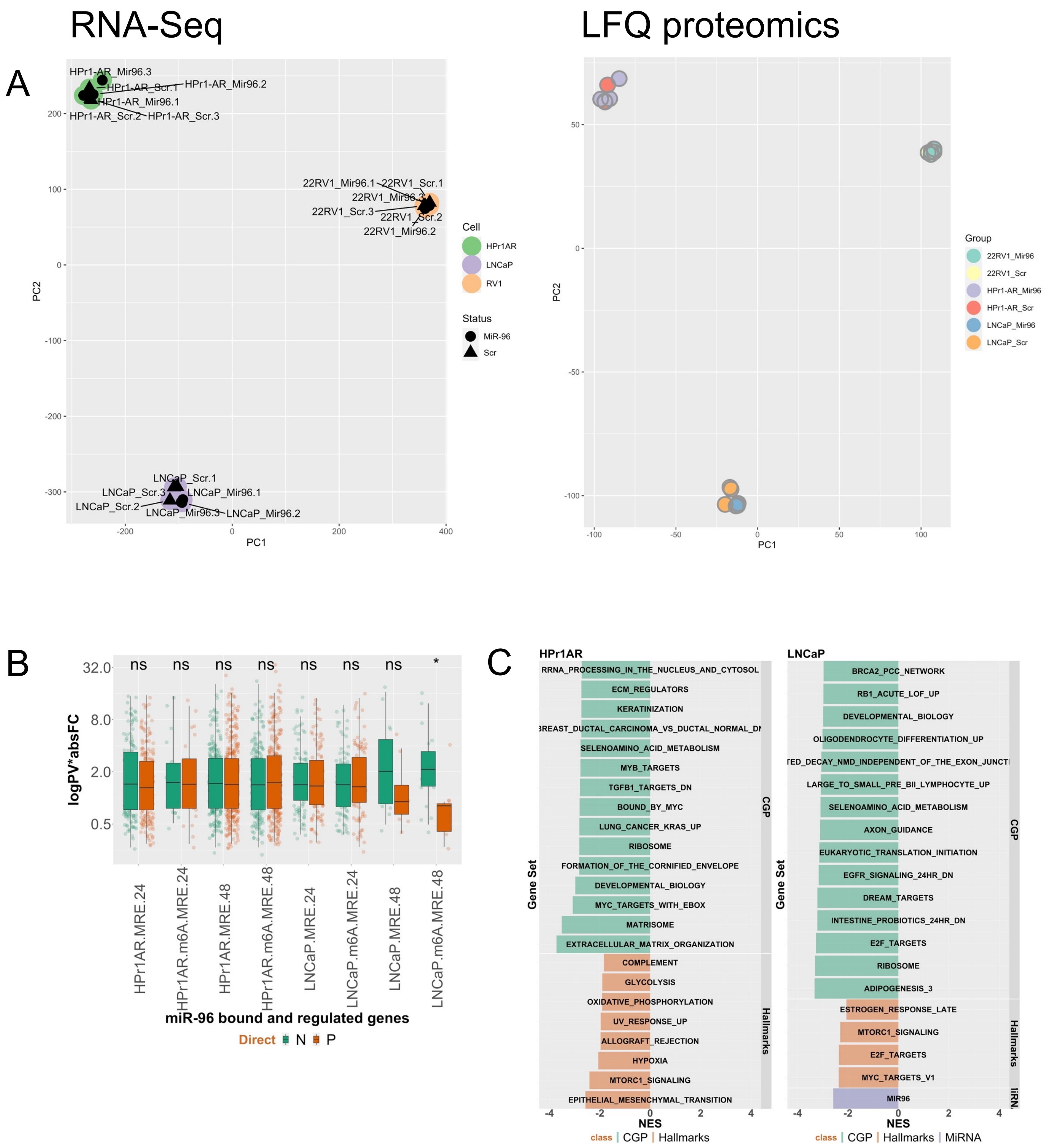

Supplementary Figure 5

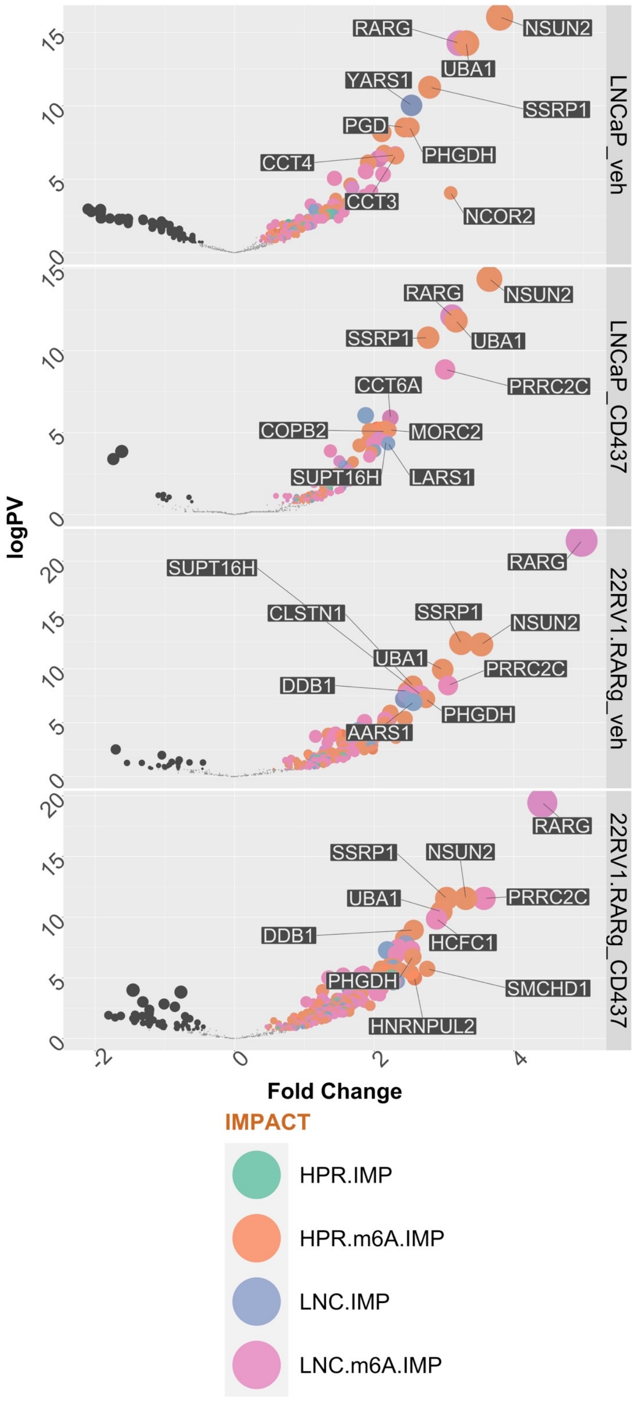

Supplementary Figure 6

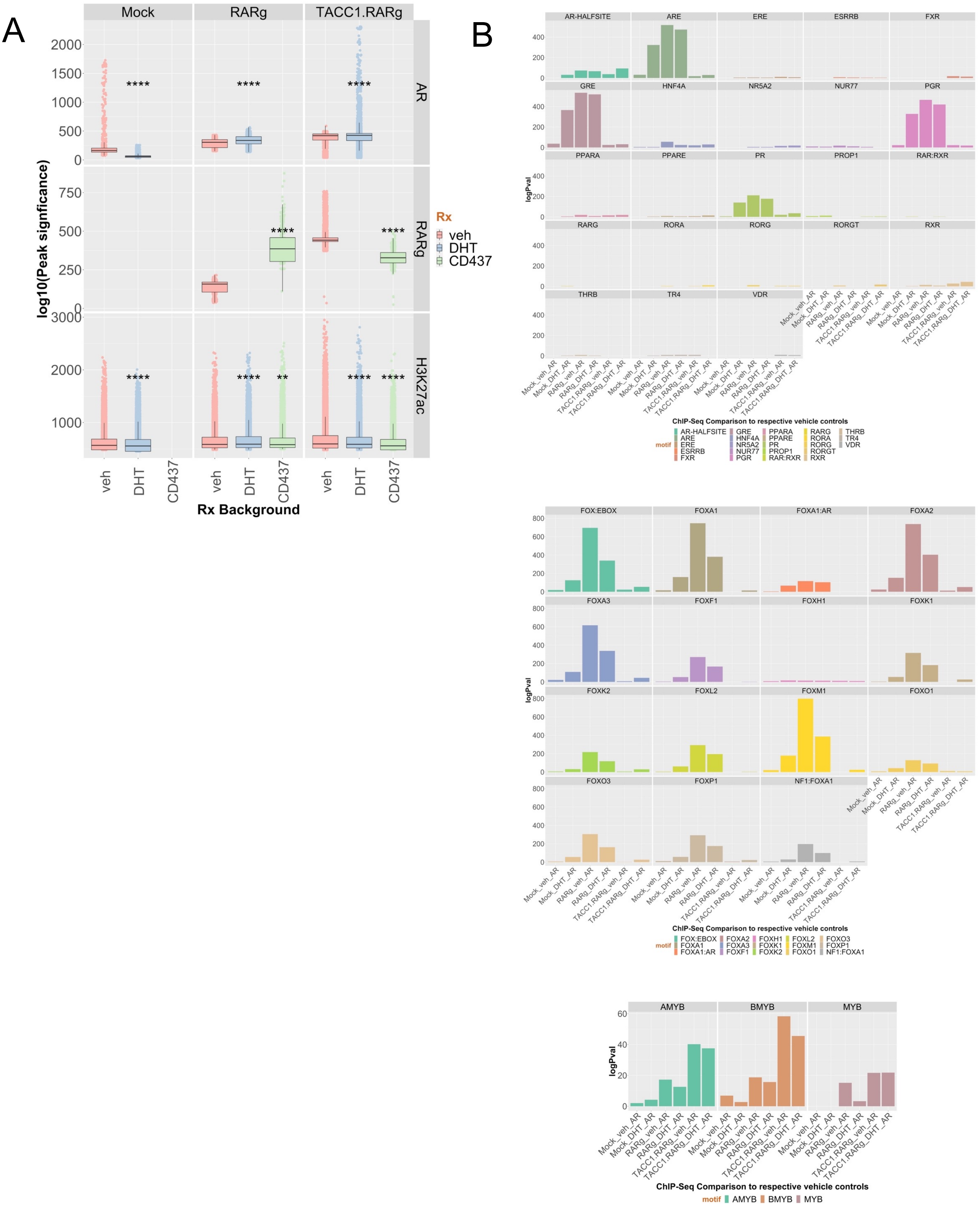

Supplementary Figure 7

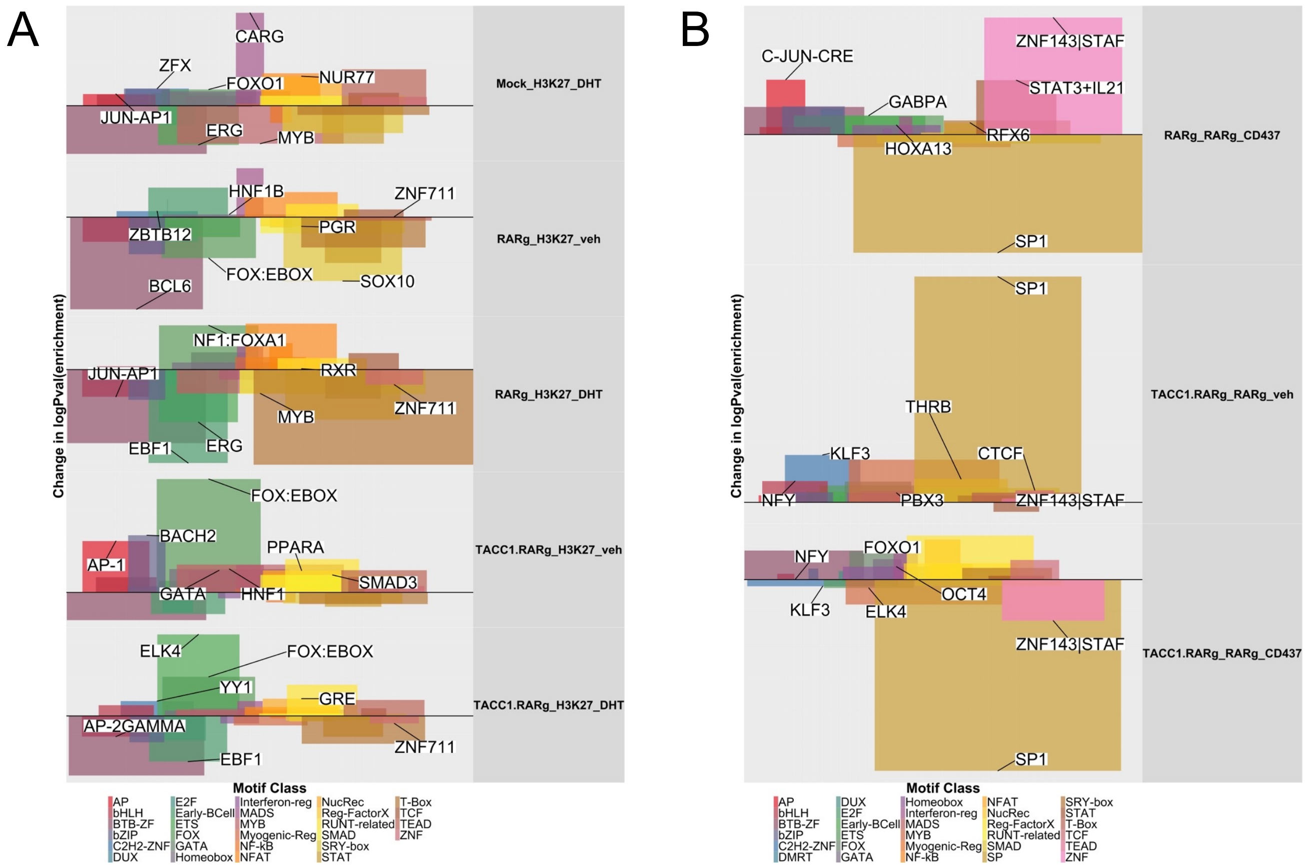

Supplementary Figure 8

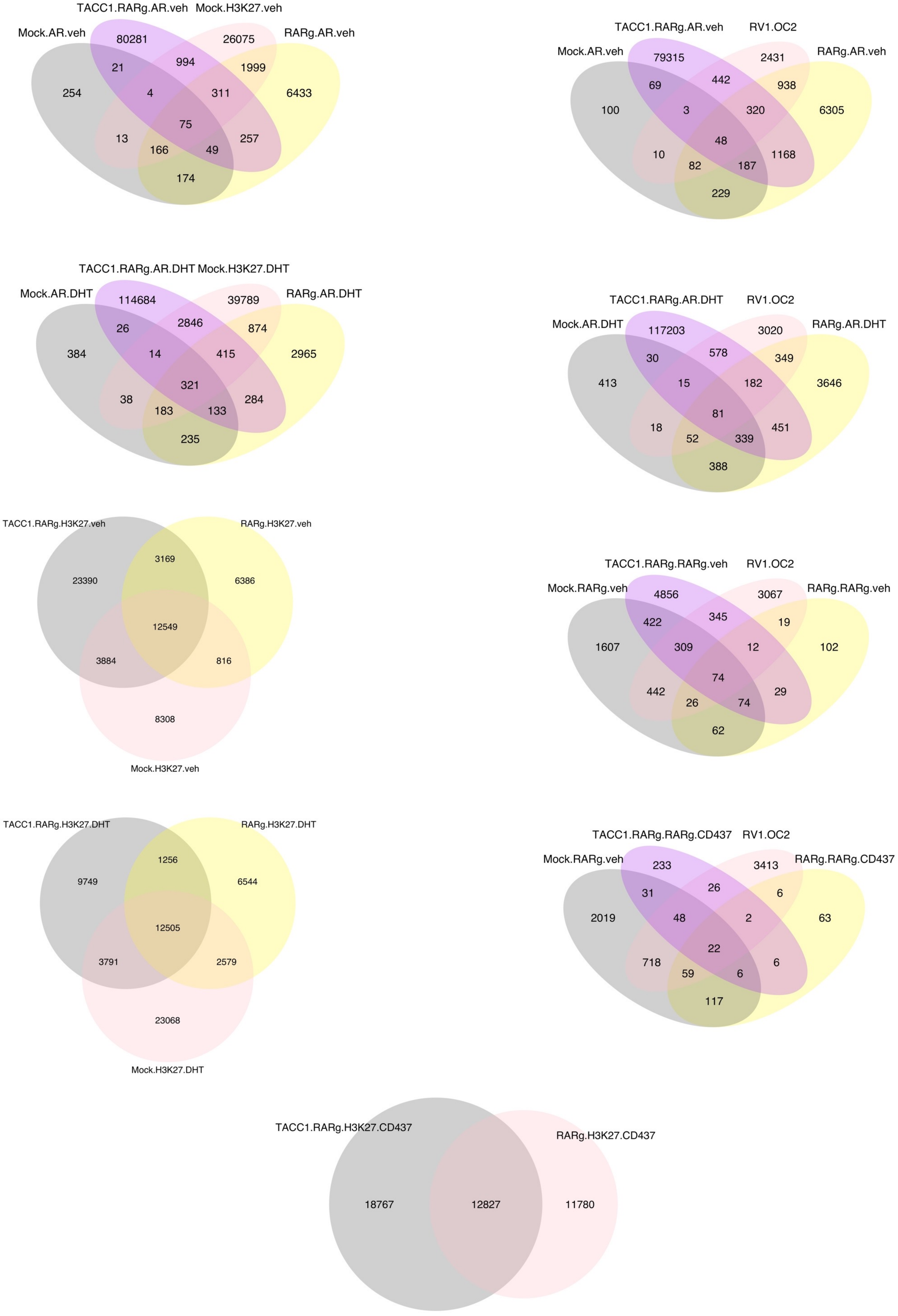

Supplementary Figure 9

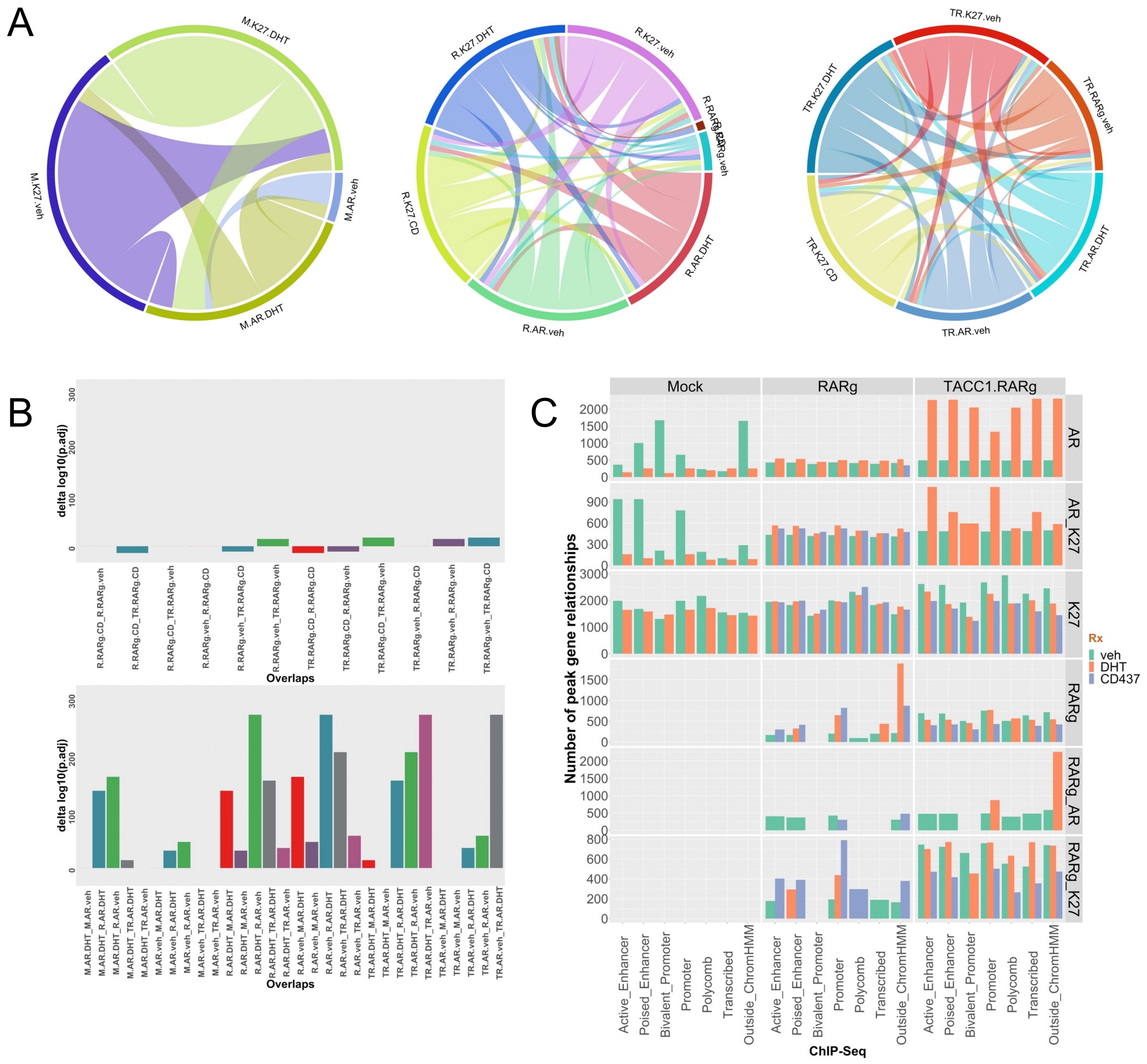

Supplementary Figure 10

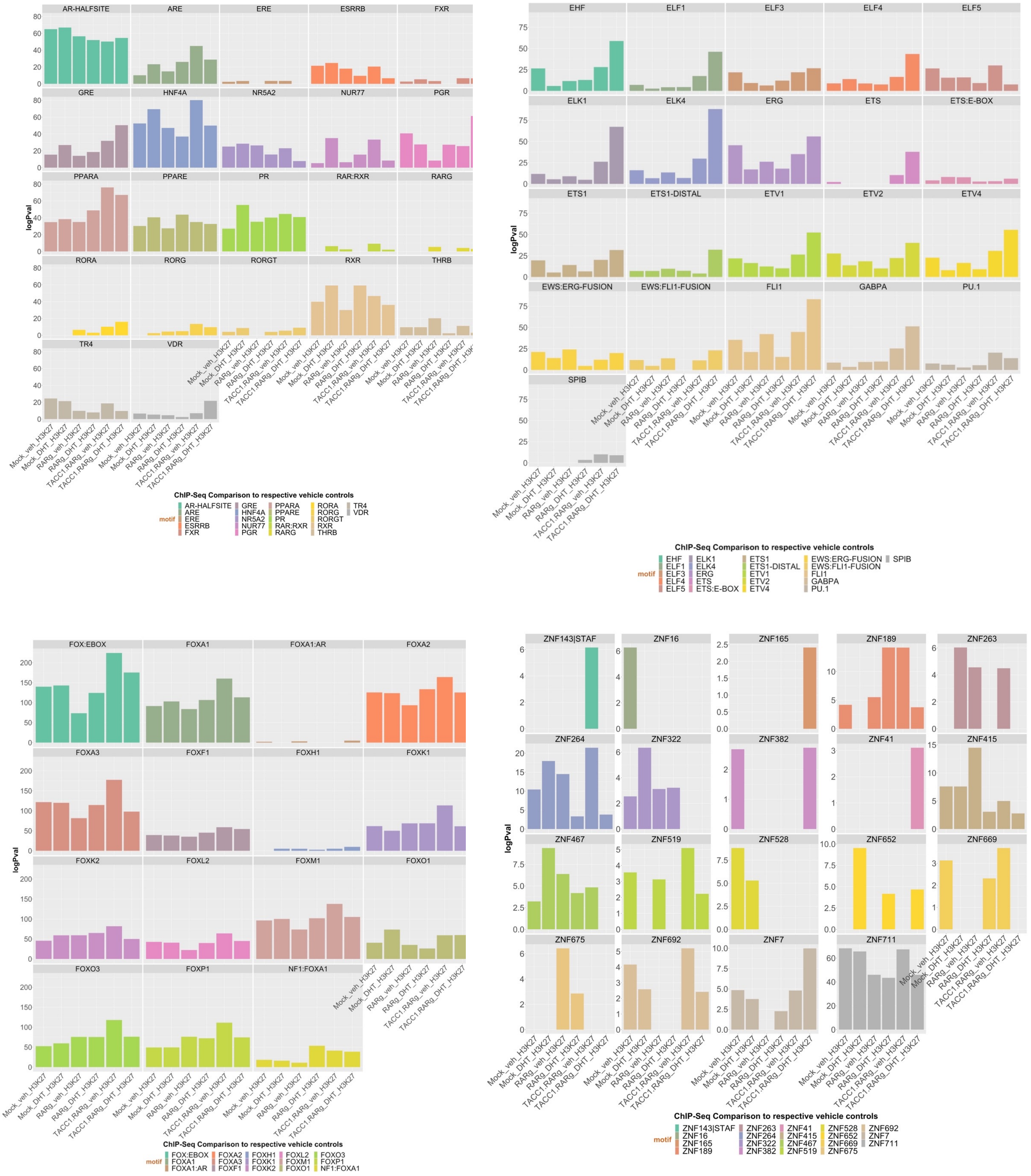

Supplementary Figure 11

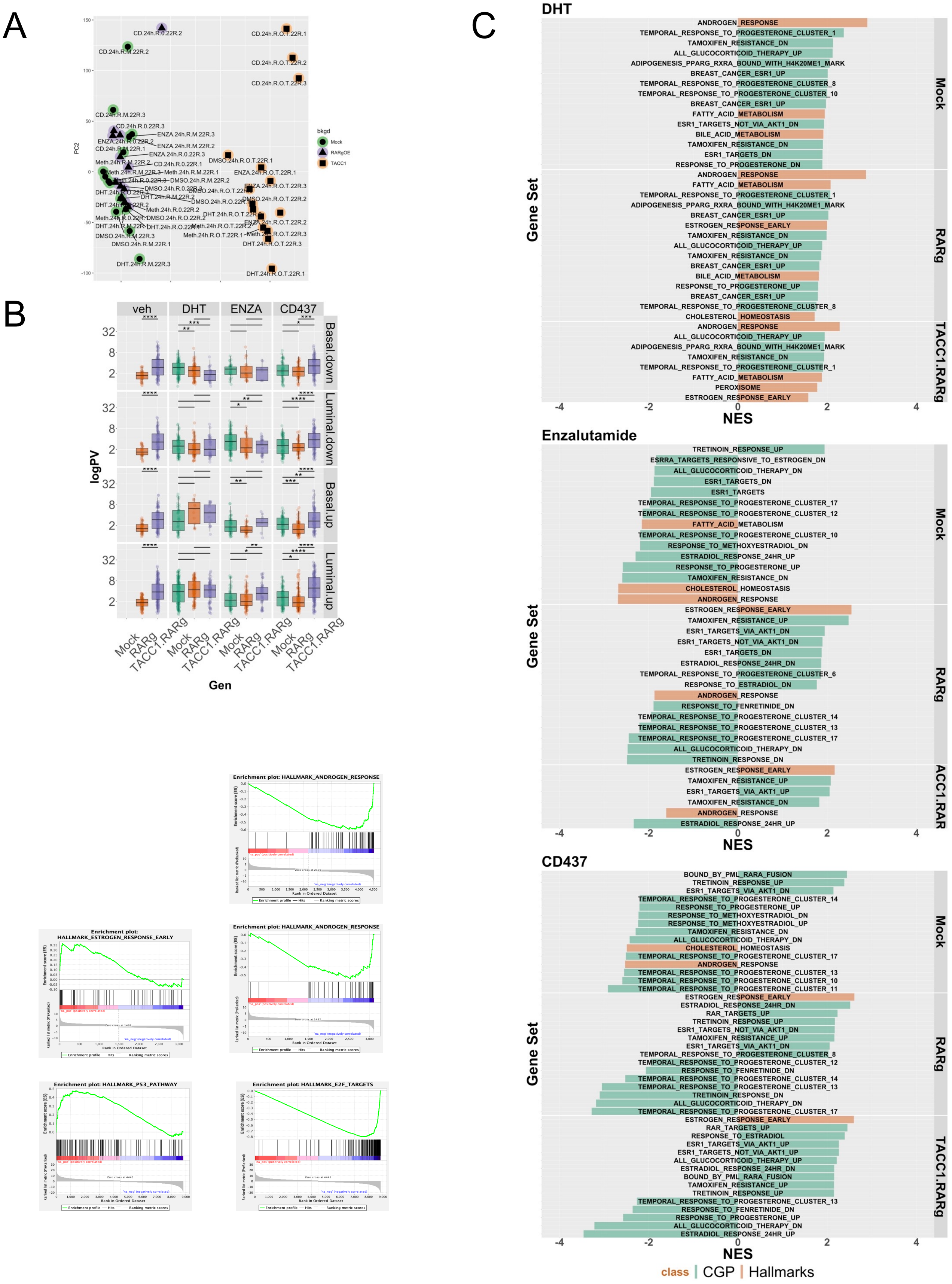

Supplementary Figure 12

A

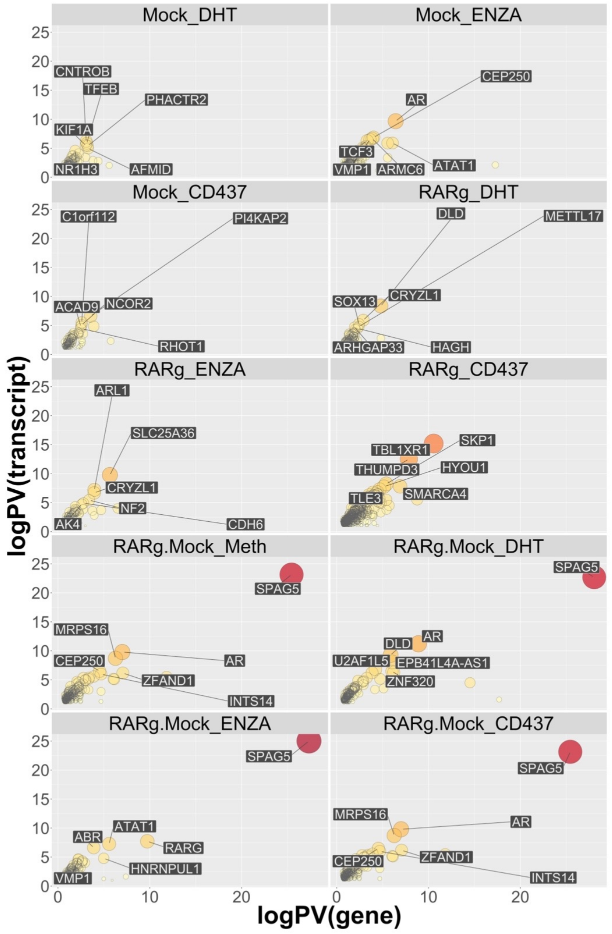

B

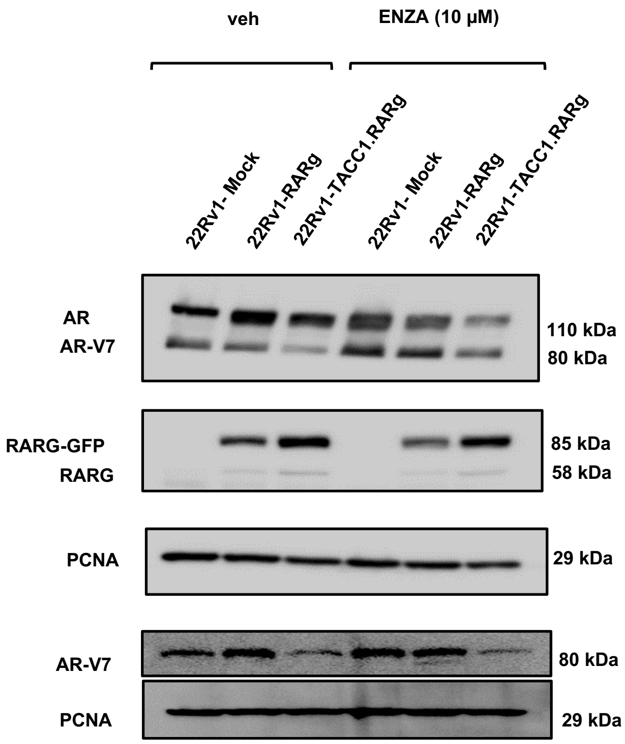

Supplementary Figure 13

A

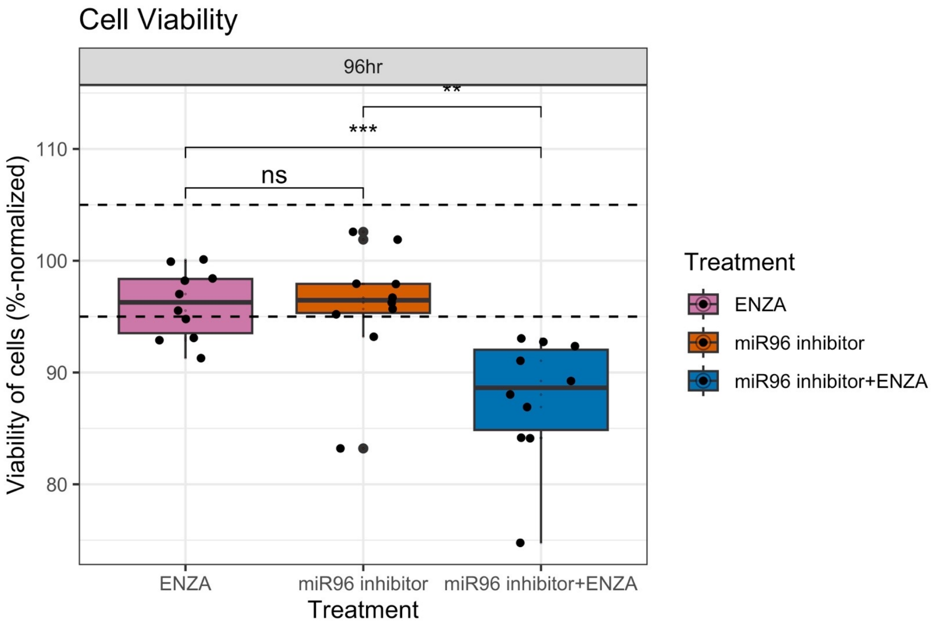

B

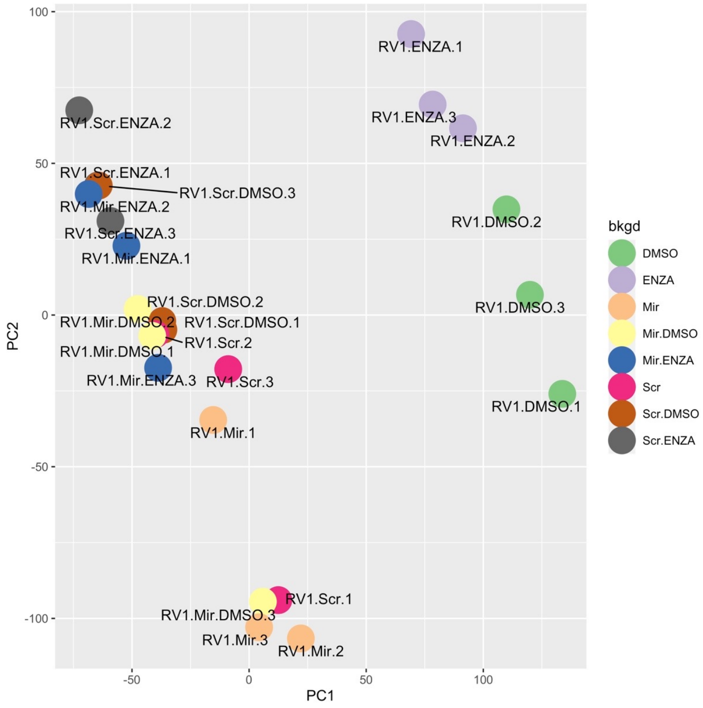

Supplementary Figure 14

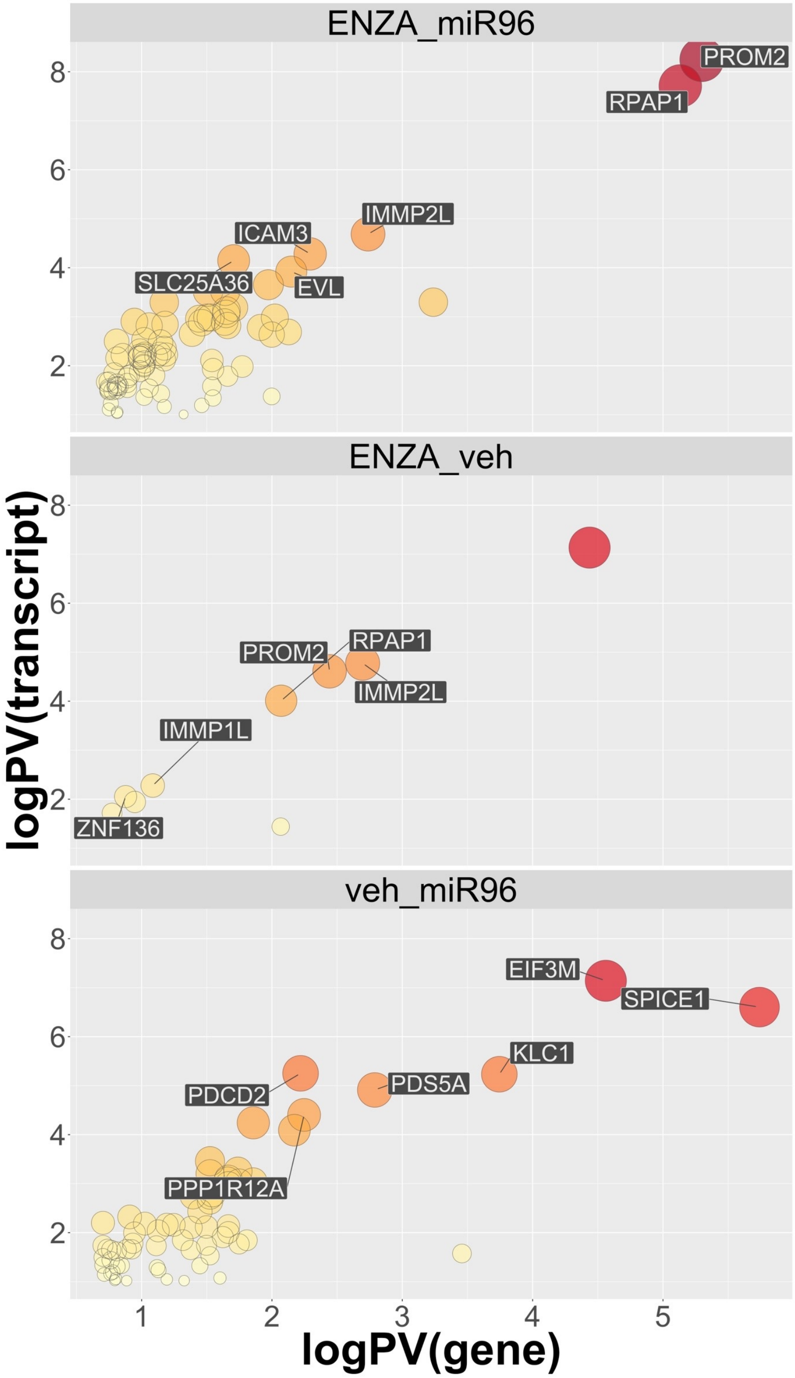

Supplementary Figure 15

A

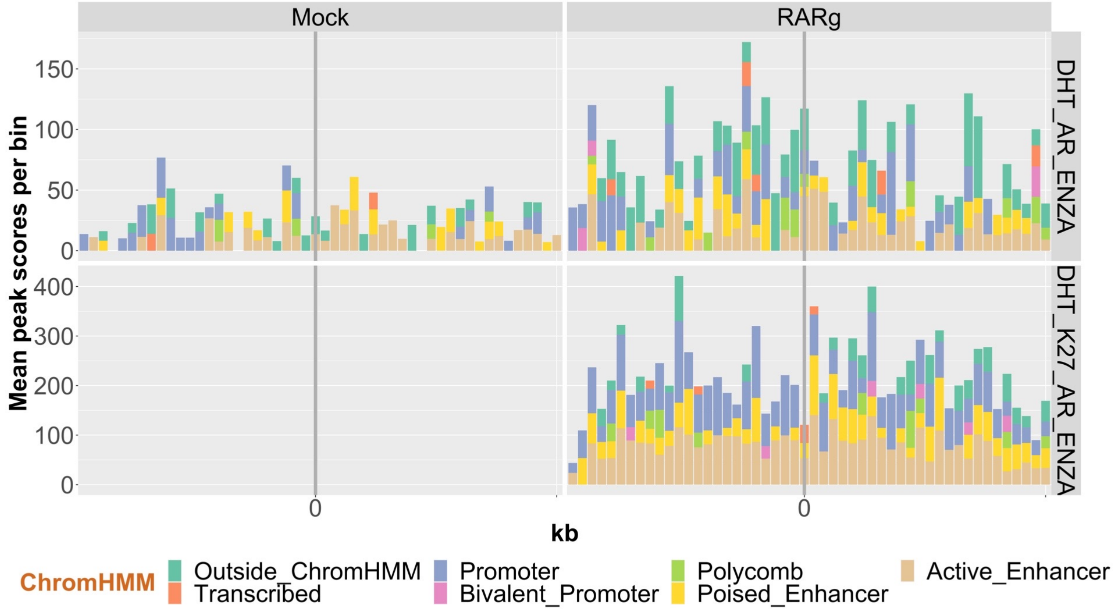

B

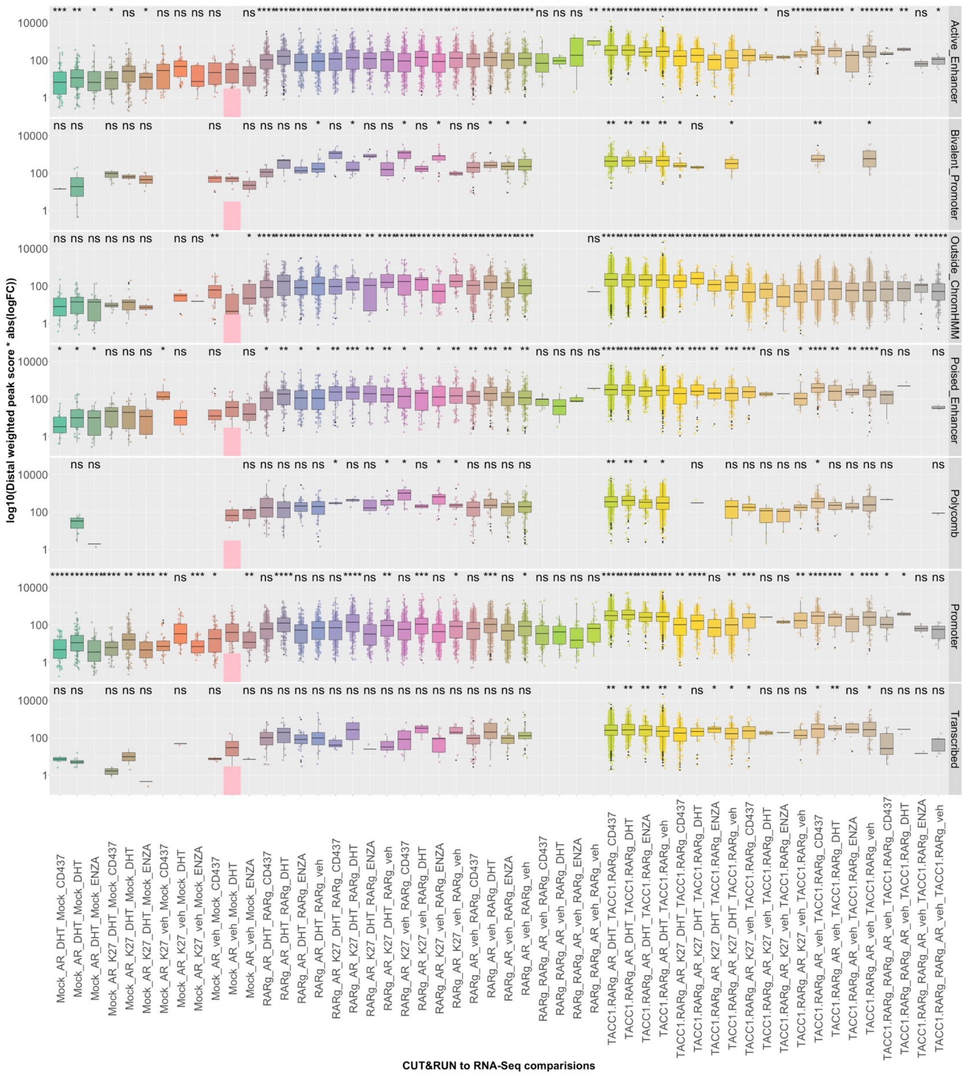

Supplementary Figure 16

A

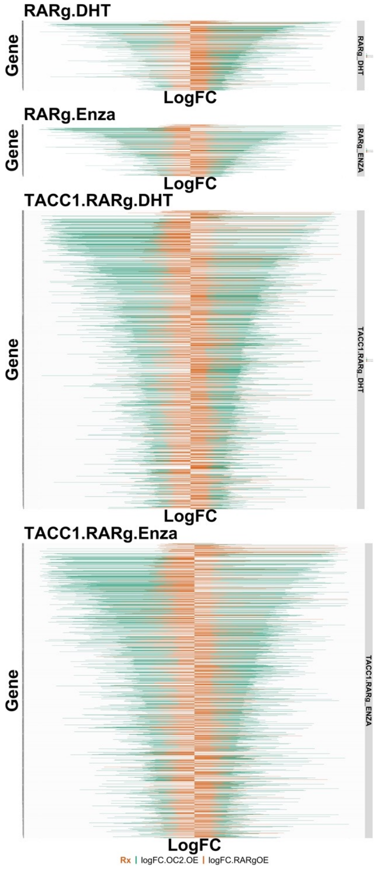

B

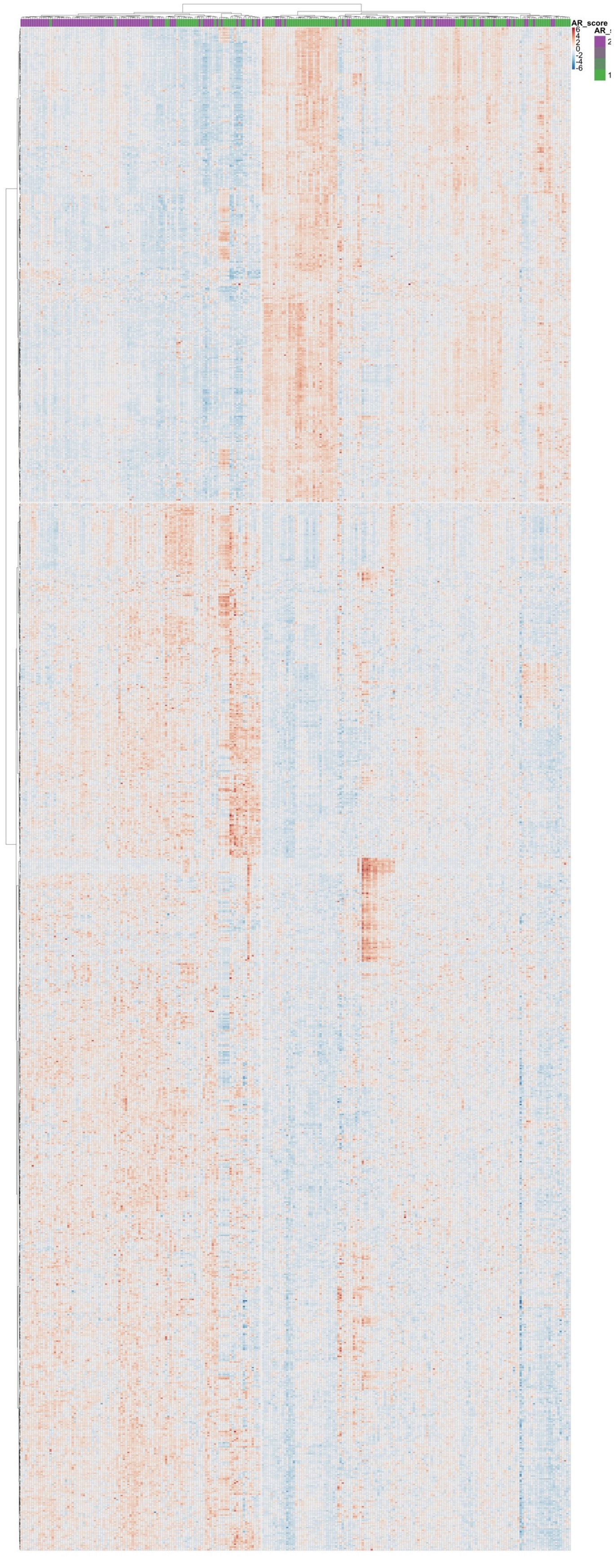

Supplementary Figure 17
